# Therapeutic radiation exposure of the abdomen during childhood induces chronic adipose tissue dysfunction

**DOI:** 10.1172/jci.insight.153586

**Published:** 2021-11-08

**Authors:** Xiaojing Huang, Olivia A. Maguire, Jeanne M. Walker, Caroline S. Jiang, Thomas S. Carroll, Ji-Dung Luo, Emily Tonorezos, Danielle Novetsky Friedman, Paul Cohen

**Affiliations:** 1 Department of Radiation Oncology, Memorial Sloan Kettering Cancer Center (MSKCC), New York, New York, USA.; 2 Laboratory of Molecular Metabolism, The Rockefeller University, New York, New York, USA.; 3 Weill Cornell/Rockefeller/Sloan Kettering Tri-institutional MD-PhD Program, New York, New York, USA.; 4 The Rockefeller University Hospital,; 5 Center for Clinical and Translational Science, and; 6 Bioinformatics Resource Center, The Rockefeller University, New York, New York, USA.; 7 Office of Cancer Survivorship, Division of Cancer Control and Population Sciences, National Cancer Institute, Rockville, Maryland, USA.; 8 Department of Pediatrics, MSKCC, New York, New York, USA.

**Keywords:** Metabolism, Oncology, Adipose tissue, Expression profiling, Macrophages

## Abstract

**BACKGROUND:**

Childhood cancer survivors who received abdominal radiotherapy (RT) or total body irradiation (TBI) are at increased risk for cardiometabolic disease, but the underlying mechanisms are unknown. We hypothesize that RT-induced adipose tissue dysfunction contributes to the development of cardiometabolic disease in the expanding population of childhood cancer survivors.

**METHODS:**

We performed clinical metabolic profiling of adult childhood cancer survivors previously exposed to TBI, abdominal RT, or chemotherapy alone, alongside a group of healthy controls. Study participants underwent abdominal s.c. adipose biopsies to obtain tissue for bulk RNA sequencing. Transcriptional signatures were analyzed using pathway and network analyses and cellular deconvolution.

**RESULTS:**

Irradiated adipose tissue is characterized by a gene expression signature indicative of a complex macrophage expansion. This signature includes activation of the *TREM2*-*TYROBP* network, a pathway described in diseases of chronic tissue injury. Radiation exposure of adipose is further associated with dysregulated adipokine secretion, specifically a decrease in insulin-sensitizing adiponectin and an increase in insulin resistance–promoting plasminogen activator inhibitor-1. Accordingly, survivors exhibiting these changes have early signs of clinical metabolic derangement, such as increased fasting glucose and hemoglobin A1c.

**CONCLUSION:**

Childhood cancer survivors exposed to abdominal RT or TBI during treatment exhibit signs of chronic s.c. adipose tissue dysfunction, manifested as dysregulated adipokine secretion that may negatively impact their systemic metabolic health.

**FUNDING:**

This study was supported by Rockefeller University Hospital; National Institute of General Medical Sciences (T32GM007739); National Center for Advancing Translational Sciences (UL1 TR001866); National Cancer Institute (P30CA008748); American Cancer Society (133831-CSDG-19-117-01-CPHPS); American Diabetes Association (1-17-ACE-17); and an anonymous donor (MSKCC).

## Graphical abstract

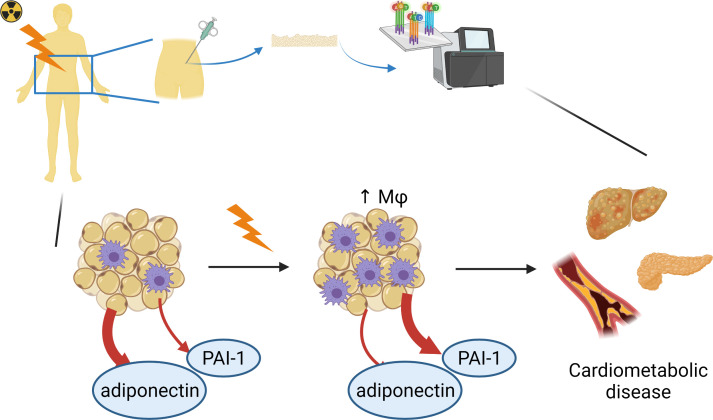

## Introduction

Through decades of concerted multidisciplinary effort, the average 5-year survival rate for all childhood cancers now exceeds 80% (1), resulting in a population of 400,000 survivors in the United States in 2019 (2). Despite this tremendous progress, childhood cancer survivors remain vulnerable to premature morbidity and mortality from treatment-related late effects (3), including diabetes mellitus (DM), dyslipidemia, and cardiovascular disease (4), with a 7-fold increased risk of cardiac death compared with the age-matched general population (5). In particular, a history of total body irradiation (TBI) or abdominal radiotherapy (RT) has been associated with an increased risk of DM (6, 7). The molecular and cellular mechanisms underlying this strong clinical association remain unknown but have been postulated to arise from RT-induced injury to endocrine organs, such as the hypothalamic-pituitary axis (8) and pancreas (9). However, another important endocrine organ, the abdominal adipose tissue, has received comparatively less consideration as a target of RT-induced injury.

Formerly seen as an inert lipid storage depot, adipose tissue is now recognized as a complex endocrine organ with a central role in systemic metabolic homeostasis (10). It displays significant regional variation in form and function, with both metabolically “protective” depots such as s.c. adipose tissue (SAT) and “unhealthy” depots such as visceral adipose tissue (VAT) (11). Consequently, RT-induced injury to the abdominal adipose depots could facilitate development of DM through disruption of their physiologic functions in maintaining whole-body energy balance. This hypothesis is supported by a small number of animal studies. TBI-treated mice exhibit reduced proliferative and adipogenic capacity in SAT (12) and develop systemic insulin resistance on high-fat diets, accompanied by decreased insulin responsiveness of preadipocytes isolated from VAT (13). TBI-treated macaques follow a similar course; notably, their SAT is characterized by increased macrophage infiltration (14), which in humans correlates with markers of poor metabolic health such as fatty liver and insulin resistance, independent of total adiposity (15).

Despite these intriguing results from preclinical models, no studies have yet examined the direct effects of radiation on human adipose tissue. We sought to address this knowledge gap by identifying molecular changes in adipose tissue from adult survivors of childhood cancer previously treated with abdominal RT or TBI, compared with individuals treated with chemotherapy only or healthy controls. We focused on relatively healthy subjects with no prior diagnosis of obesity or DM to determine if early changes could be detected that might later be validated as biomarkers for eventual progression to overt DM or as targets for therapeutic interventions that might mitigate this pathology.

## Results

### Clinical and metabolic characteristics of study subjects.

**Figure 1:**
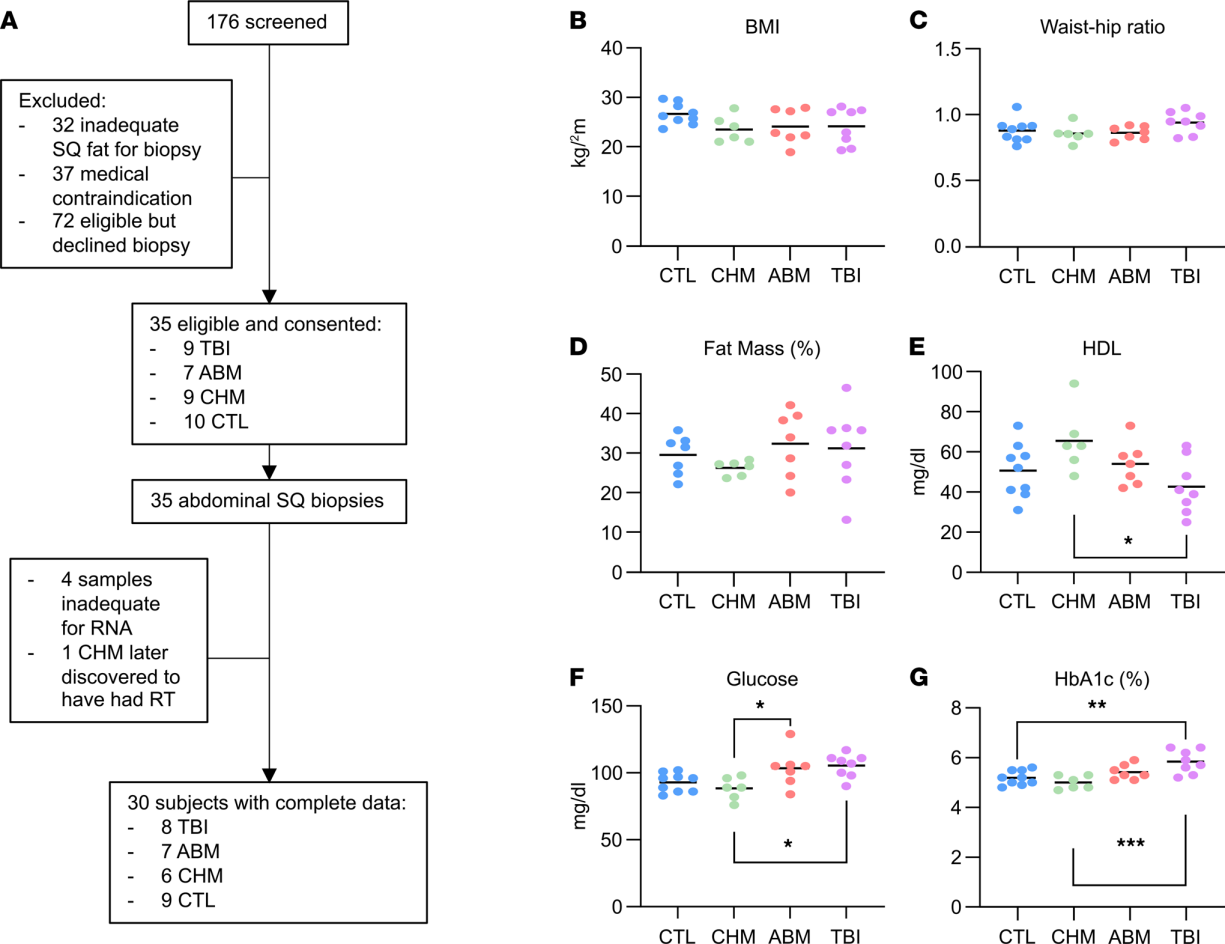
Study schematic and participant characteristics. (**A**) Participant flow diagram. (**B**–**G**) BMI, waist/hip ratio, fat mass %, fasting serum high density lipoprotein (HDL), fasting serum glucose, and glycated hemoglobin (HbA1c) measurements in CTL (*n* = 9, except **D**, which is missing 2 subjects), CHM (*n* = 6), ABM (*n* = 7), and TBI (*n* = 8) groups. *P < 0.05; **P < 0.01; ***P < 0.001 on 1-way ANOVA with Tukey’s multitest correction for post hoc pairwise comparisons.

We recruited 35 adult participants (≥18 years) in 4 categories: (a) survivors of childhood cancer with prior history of TBI, (b) survivors previously exposed to abdominal (ABM) RT, (c) survivors treated with chemotherapy (CHM) only without any prior RT exposure, and (d) healthy volunteers (CTL) with no prior cancer diagnosis. Participants were recruited from the MSKCC Long-Term Follow-Up Clinic (survivors) and the Rockefeller University Hospital (controls). All survivors received chemotherapy as part of their treatment. One CHM subject was later excluded after discovery of prior neck/mediastinal RT, while 4 subjects did not have RNA sequencing (RNA-seq) data due to inadequate biopsy material. This resulted in a study cohort of 30 subjects (*n* = 8 in TBI group, 7 in ABM, 6 in CHM, 9 in CTL) with clinical metabolic profiling data and RNA-seq results from an abdominal SAT biopsy (Figure 1A). The clinical characteristics of each group are summarized in Table 1, with additional details of treatment histories in Supplemental Table 1 (supplemental material available online with this article; https://doi.org/10.1172/jci.insight.153586DS1). Overall, the CHM group completed cancer treatment significantly more recently than the ABM and TBI groups and tended to be younger, while RT dose was higher in the ABM group compared with TBI, as expected given the characteristic differences between these treatment regimens. The TBI group was notably enriched for subjects with a history of allogeneic BM transplant (BMT).

The groups were not significantly different from one another on standard anthropometric surrogates for metabolic health such as BMI, waist/hip ratio (WHR), weight, and percent lean or fat mass as assessed by air displacement plethysmography (Figure 1, B–D, and Supplemental Figure 1, A and B). Dyslipidemia was observed in one participant in the TBI group with abnormally high triglycerides (Supplemental Figure 1C); that participant also had the lowest HDL (Figure 1E), which was overall significantly lower in the TBI group (42.6 ± 13.6 versus 65.5 ± 15.7 mg/dL for CHM group, adjusted *P* = 0.014). LDL and total cholesterol were not significantly different among groups (Supplemental Figure 1, D and E). The ABM and TBI groups had significantly higher fasting glucose levels (103.4 ± 13.8 and 105.4 ± 8.7 mg/dL, respectively) compared with CHM participants (88.3 ± 8.3 mg/dL; Figure 1F, adjusted *P* < 0.05 for both comparisons), while the TBI group had significantly higher HbA1c levels (5.84% ± 0.47%) compared with the CHM (5.00% ± 0.27%, *P* < 0.001) and CTL (5.19% ± 0.29%, *P* < 0.01) groups. Furthermore, 5 of 9 participants in the TBI group and 2 of 7 in the ABM group were prediabetic (HbA1c ≥ 5.7%), while no participants in either CTL or CHM groups were prediabetic (Figure 1G, *P* < 0.01 for Fisher’s exact test comparing RT to non-RT participants). High-sensitivity C-reactive protein, a marker of atherosclerotic cardiovascular disease risk (16), was in the low-risk range (<2.0 mg/L) for all participants (Supplemental Figure 1F). Given the dependency of many metabolic outcomes on age and the trend toward younger age in the CHM group, we performed ANCOVA to compare the group differences observed in HDL, fasting glucose, and HbA1c while controlling for age. HDL was no longer significantly different across the groups after adjusting for age (*P* = 0.079 for group effect, *P* = 0.014 for age effect); however, fasting glucose (*P* = 0.011 for group, *P* = 0.023 for age) and HbA1c (*P* = 0.00046 for group, *P* = 0.0044 for age) retained their differences across groups after adjusting for age. Post hoc pairwise comparisons with Tukey’s multitest correction showed significant differences in HbA1c between the TBI group and either the CHM (*P* = 0.002) or CTL groups (*P* = 0.0007). Post hoc pairwise comparisons for glucose did not reveal any significant between-group differences. Similar results were obtained with ANCOVA for group and time since treatment for the CHM, ABM, and TBI groups, although fasting glucose also lost between-group significance on this analysis in addition to HDL. HbA1c differences among the 3 survivor groups remained significant (*P* = 0.013 for group; *P* = 0.15 for time since treatment), with post hoc pairwise comparisons showing a significant difference between CHM and TBI groups after Tukey’s multitest correction (*P* = 0.029).

### Gene expression signatures of irradiated human SAT.

**Figure 2:**
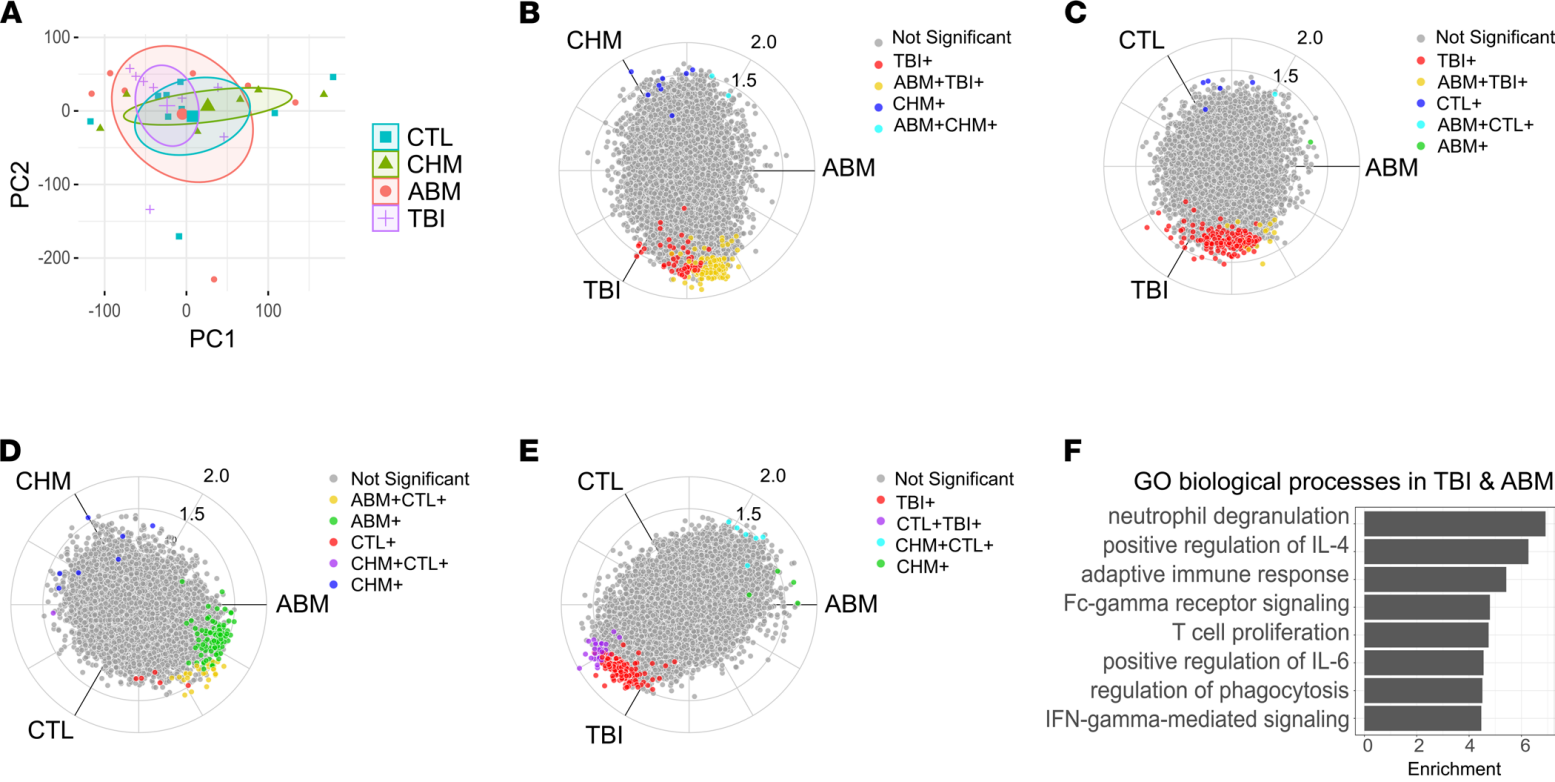
Gene expression signatures of irradiated adipose tissue. (**A**) Principal component analysis of RNA-seq results. Ellipses show 95% CI. (**B**–**E**) Polar-coordinate volcano plots comparing TBI, ABM, CHM; TBI, ABM, CTL; ABM, CHM, CTL; and TBI, CHM, CTL groups. Individual genes are represented as points. Radial coordinates of each gene are calculated based on the gene’s expression *Z* score in each of the subject groups labeled on the radial axes; proximity to an axis indicates increased expression in the group indicated on that axis. Colored points indicate significantly upregulated genes in the labeled subject groups; significance is determined by a *P* value cutoff of 0.01 using the likelihood ratio test implemented in DESeq2. (**F**) Gene ontology (GO) enrichment analysis of genes upregulated in both TBI and ABM groups, scored as –log_10_ of the adjusted *P* value from topGO.

To gain insight into the molecular pathways that characterize irradiated adipose tissue, we isolated and bulk sequenced RNA from needle aspirations of SAT from the right or left lower abdominal quadrants of each participant (Supplemental Table 1). Principal component analysis (PCA) of all 23,434 transcripts showed no separation of the subject groups (Figure 2A), likely reflecting the heterogeneity of our study cohort. Nevertheless, differential gene expression analysis using DESeq2 (17) uncovered a number of significant changes in the TBI group compared with the CTL (74 genes) and CHM (1191) groups (Supplemental Figure 2, A and B), while the ABM group had significantly fewer differences compared with these 2 groups (2 and 36, respectively; Supplemental Figure 2, C and D). This discrepancy between the TBI and ABM groups may be due to a significant portion of the ABM cohort (5 of 7) being treated before 2000 or at other institutions, thus precluding the ability to ensure that the abdominal SAT biopsy site coincided with the RT field.

Given the large number of pairwise comparisons with 4 subject groups, we used a polar coordinate-based method (18) to visualize differentially expressed genes (DEGs) in 3-way comparisons using the likelihood ratio test for statistical significance (Figure 2, B–E). We observed some overlap in genes upregulated in both the TBI and ABM groups compared with the CHM group (Figure 2B) or, to a lesser extent, the CTL group (Figure 2C). We also noted that the majority of all differences observed were driven by the TBI and ABM groups, with few differences between the CTL (Figure 2, C–E) and CHM (Figure 2, B, D, and E, and Supplemental Figure 2F) groups; there were also few differences between the TBI and ABM groups (Supplemental Figure 2E). The genes upregulated in both the TBI and ABM groups (Supplemental Table 2) are enriched for immune-related terms by gene ontology (GO) analysis, including positive regulation of production of the cytokines IL-4, IL-6, and IFN-γ and myeloid processes such as neutrophil degranulation and phagocytosis (Figure 2F and Supplemental Table 3). GO analysis of the genes upregulated in the TBI or ABM groups, but not necessarily both, produced similar results (Supplemental Figure 2G).

### Weighted gene correlation network analysis (WGCNA).

**Figure 3:**
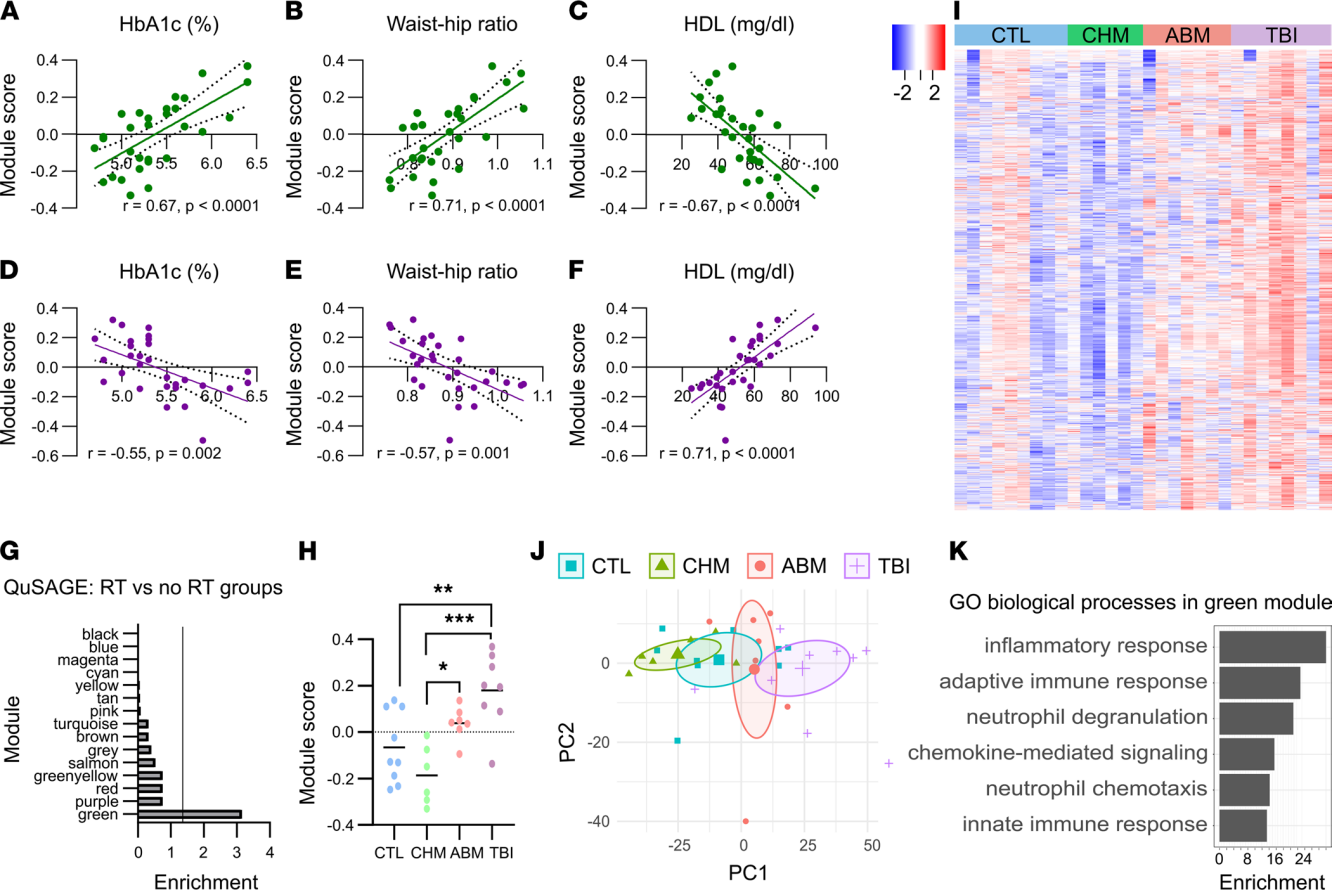
WGCNA modules associate with metabolic health. (**A**–**C**) Correlation of green module expression with HbA1c, waist/hip ratio, and HDL across all subjects. (**D**–**F**) Correlation of purple module expression with HbA1c, waist/hip ratio, and HDL across all subjects. (**G**) QuSAGE enrichment scores (–log_10_ of the FDR-adjusted *P* values) for each WGCNA module in the comparison of subjects who received RT (TBI and ABM groups) with those who did not (CTL and CHM groups). Cutline corresponds to *P* = 0.05. (**H**) Green module scores (calculated from WGCNA’s eigengene function; *P < 0.05, **P < 0.01, ***P < 0.001 on 1-way ANOVA with Tukey’s multitest correction for post hoc pairwise comparisons). (**I**) Heatmap of green module gene expression. (**J**) Principal component analysis of gene expression matrix restricted to only green module genes. Ellipses show 95% CI. (**K**) Gene ontology (GO) enrichment of green module genes, scored as –log_10_ of the adjusted *P* value from topGO.

Constraining RNA-seq analysis to the evaluation of individual DEGs can miss smaller but coordinated changes across biological pathways or networks. To circumvent this limitation, we employed an unbiased approach using WGCNA (19) to identify groups or modules of genes displaying correlated expression across all samples (Supplemental Table 4). We then correlated module expression scores for each study subject with their clinical traits (Supplemental Figure 3A). This analysis revealed 2 modules (green and purple) with highly significant correlations with HbA1c, WHR, and HDL. The green module was positively correlated with a profile of worse metabolic health, while the purple module was correlated with improved metabolic health (Figure 3, A–C), and it revealed the opposite finding for the purple module (Figure 3, D–F). However, when we used quantitative set analysis of gene expression (QuSAGE) (20) to determine which modules were enriched in participants who had received RT compared with those who had not, only the green module was significantly upregulated in the former (*P* < 0.001; Figure 3G). Expression scores for the green module were significantly higher in the TBI group compared with the CTL and CHM groups, and they were higher in the ABM group compared with the CHM group (Figure 3, H and I), while no differences among groups were observed for the purple module (Supplemental Figure 3B). When PCA is restricted to just the 1347 green module genes, the TBI and ABM groups separate from CHM and CTL along PC1 (Figure 3J), indicating the ability of this module to discriminate among the different groups.

Similar to results obtained with the DEGs identified by DESeq2, GO analysis of green module genes also produced terms related to inflammatory pathways (Figure 3K and Supplemental Table 5). Indeed, there was overrepresentation of DEGs in the green module compared with other modules (Supplemental Table 6). In contrast, the purple module was enriched for GO terms related to fatty acid catabolism (Supplemental Figure 3C). Since this module could not distinguish between RT and non-RT participants, we investigated whether a confounding variable was driving its expression pattern. Age is a well-known risk factor for metabolic syndrome and, thus, a possible determinant of both green and purple module expression, given their association with a worse and an improved metabolic profile, respectively. ANCOVA examining the effect of group on green module expression while controlling for age showed a highly significant group effect (*P* = 0.00047) but a more modest age effect (*P* = 0.045), while purple module expression showed a significant effect for age (*P* = 0.00075) but not for group (*P* = 0.32). In addition to age, BMT status and time since treatment are also potential confounders. ANCOVA models examining the effect of group and time since treatment or group and BMT status on green module scores showed significant effects for group (*P* = 0.0027 in model with group and time since treatment; *P* = 0.0017 in model with group and BMT status) but not time since treatment (*P* = 0.34) or BMT status (*P* = 0.69) in the subset of childhood cancer survivors.

The top green module genes identified by QuSAGE include a number of macrophage markers such as *SPP1*, *MMP9*, *TREM2*, *ACP5*, *ALCAM,* and *VSIG4* (Supplemental Figure 4A and Supplemental Table 7). Interestingly, both proinflammatory (*SPP1*, *MMP9*; refs. 21, 22) and immunomodulatory (*VSIG4*, *TREM2*; refs. 23, 24) signals are present. The transcriptional profile of a number of these genes (*SPP1*, *MMP9*, *ACP5*, *ALCAM*, *VSIG4*), as well as several identified as DEGs by DESeq2 (*FCER1G*, *HAVCR2*, *HLA-DRA,* *LGMN*, *C1QB*) was recapitulated by quantitative PCR (qPCR) (Supplemental Figure 4B, *P* = 0.0047 for group effect on rank-transformed mixed effects model), with upregulation across all genes in the TBI group relative to the CHM (FDR-adjusted post hoc *P* = 0.0017) and CTL groups (adjusted *P* = 0.008) and in the ABM group relative to the CHM (adjusted *P* = 0.04) but not the CTL group (adjusted *P* = 0.22). In contrast, levels of adipogenesis-related genes *PPARG*, *ADIPOQ*, and *FABP4* were unchanged across subject groups (*P* values from 0.11 to 0.992 on Kruskal-Wallis test) (Supplemental Figure 4C).

### Single-cell deconvolution reveals macrophage enrichment in irradiated adipose tissue.

**Figure 4:**
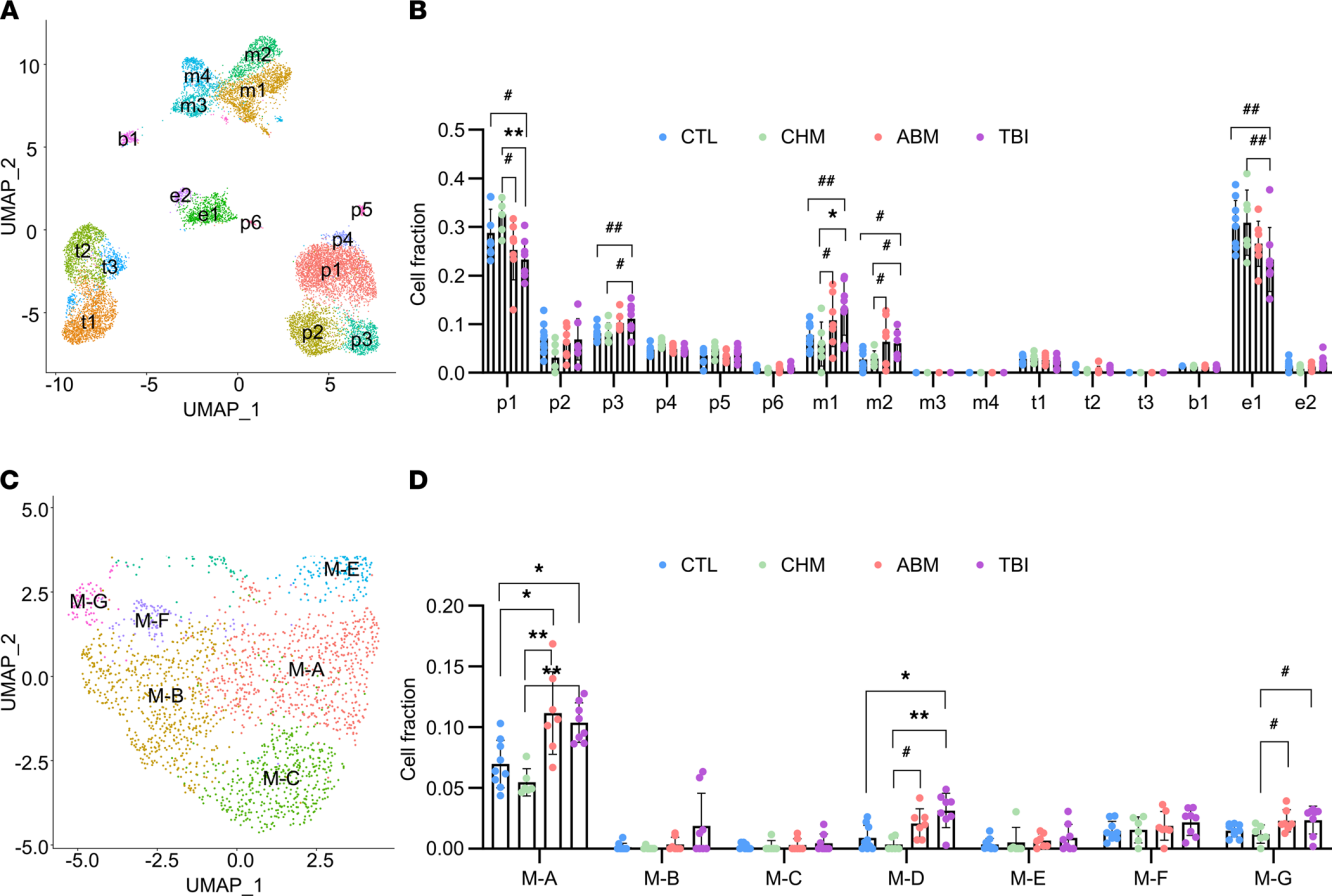
Cell type deconvolution. (**A**) UMAP of Seurat clustering of SAT cells from Vijay et al. (25). *p*, progenitor; *m*, myeloid; *t*, T cell; *b*, B cell; *e*, endothelial clusters. (**B**) CIBERSORTx deconvolution of bulk RNA-seq data using signature matrix derived from clusters in **A**. (**C**) Subclustering of *m1* and *m2* macrophage populations. (**D**) Deconvolution using signature matrix with *m1* and *m2* clusters expanded to the subclusters identified in **C**. Differences among groups in **B** were tested using repeated-measures ANOVA after aligned rank transformation, which showed significant interaction between group and cell type (*P* = 0.0003). FDR-adjusted *P* values for post hoc pairwise comparisons were calculated using estimated marginal means on only group comparisons within each cell type, not all possible pairwise comparisons within the data. Differences among groups in **D** were tested using the Kruskal-Wallis test, followed by the Dunn test for post hoc comparisons with FDR adjustment. ^#^*P* < 0.05, ^##^*P* < 0.01 on post hoc pairwise comparisons without FDR adjustment; *P < 0.05, **P < 0.01 on post hoc pairwise comparisons with FDR adjustment. Data are shown as mean ± SD.

Given the observed GO enrichments in inflammatory pathways, we hypothesized that an immune population in irradiated adipose tissue, likely composed of macrophages, was the cellular origin of this gene expression signature. We used previously published single-cell RNA-seq (scRNA-seq) data from SAT of obese nondiabetic and diabetic patients (25) and the CIBERSORTx (26) algorithm to deconvolute our bulk RNA-seq data into cell type fractions. To better match the origin of our RNA samples, we reclustered the scRNA data set using only cells originating from SAT, excluding VAT cells included in that study (Figure 4A and Supplemental Table 8). We used this clustering in CIBERSORTx to calculate the fractions of each cell type in our adipose samples (Figure 4B). Notably, we observed a trend toward enrichment of myeloid clusters *m1* and *m2* in the ABM and TBI groups, with a concomitant decrease in the fraction of adipose progenitor cells *p1* and endothelial cells *e1* (*P* = 0.0003 for interaction between participant group and cell type on repeated-measures ANOVA after aligned rank transformation; post hoc pairwise comparisons are given in Supplemental Table 9). Both *m1* and *m2* clusters are enriched for CD68^+^ cells and are identified as macrophages by SingleR annotation (Supplemental Table 8). They are also enriched for several genes of interest found by WGCNA/QuSAGE or DESeq2 (representative genes shown in Supplemental Figure 5, A and B), suggesting that adipose tissue–associated macrophages are the origin of the inflammatory gene expression signature described above. Although there was a decrease in the *p1* and *e1* fractions in the TBI and ABM groups, our previous DEG analyses did not reveal any markers mapping to these clusters; thus, we could not conclude whether this change represented an absolute decrease in cell numbers or merely a relative decrease due to expansion of the macrophage population. Intriguingly, there was also a trend toward increases in the *p3* fraction in the ABM and TBI groups (Figure 4B and Supplemental Table 9). This progenitor cluster expresses a number of collagen-related genes and is annotated as fibroblasts by SingleR (Supplemental Table 8). Tissue fibrosis is a commonly observed chronic effect of RT exposure at therapeutic doses (27), raising the possibility that enrichment of the *p3* cluster in the TBI and ABM groups represents expansion of a fibroblast-like population in irradiated adipose tissue. Moreover, fibrosis has been implicated in the development of adipose tissue dysfunction, especially when facing the challenge of caloric excess (28), and thus could represent a causal mechanism underlying RT-induced adipose injury.

To further characterize the enriched macrophage population, we subclustered *m1* and *m2* cells into 7 new groups, labeled *M-A*, *M-B, M-C, M-D, M-E, M-F*, and *M-G* (Figure 4C and Supplemental Table 10). We repeated CIBERSORTx deconvolution by substituting these subclusters for the original *m1* and *m2* clusters. The results (Figure 4D) show a significant enrichment of *M-A* and a trend toward enrichment of *M-D* and *M-G* macrophages in the TBI and ABM groups (post hoc pairwise comparisons from Kruskal-Wallis test given in Supplemental Table 11). Cluster *M-A* corresponds to a largely inflammatory phenotype expressing several of the top significant genes identified in this study, including *MMP9*, *ACP5*, and *ITGB2*, as well as *ITGAX* (CD11c) and *CD9*, markers of crown-like structure macrophages (Supplemental Figure 5C) (29, 30). However, it also expresses a number of immunomodulatory markers such as *TREM2* and galectins 3 and 9 (Supplemental Figure 5C) (24, 31, 32), suggesting a more complex phenotype beyond promoting inflammation. Notably, clustering at a higher resolution did not separate these markers into different cell clusters, indicating that the coexpression of these markers in *M-A* is not an artifact of underclustering (Supplemental Figure 5D). Clusters *M-D* and *M-G* express *SEPP1* and *MS4A6A* (Supplemental Figure 5E), macrophage markers that have been studied in a variety of contexts including atherosclerotic plaque formation (33), tumor infiltration (34, 35), and Alzheimer’s disease (36). *M-D* is additionally marked by specific expression of *FCGBP*, a differentially regulated gene in our data set (Supplemental Tables 2 and 7), and *GPR34*, a relatively uncharacterized lysophospholipid receptor (37), while *M-G* is specified by expression of CD206 (*MRC1*), which has been associated with inhibition of preadipocyte growth and differentiation (38) (Supplemental Figure 5E). The enrichment of diverse macrophage phenotypes in RT-exposed adipose tissue suggests a dynamic remodeling of the tissue, even decades after the initiating event (mean time since RT 23.2 ± 9.8 years in TBI and ABM groups).

### Impaired adipokine secretion in chronic adipose tissue response to RT.

**Figure 5:**
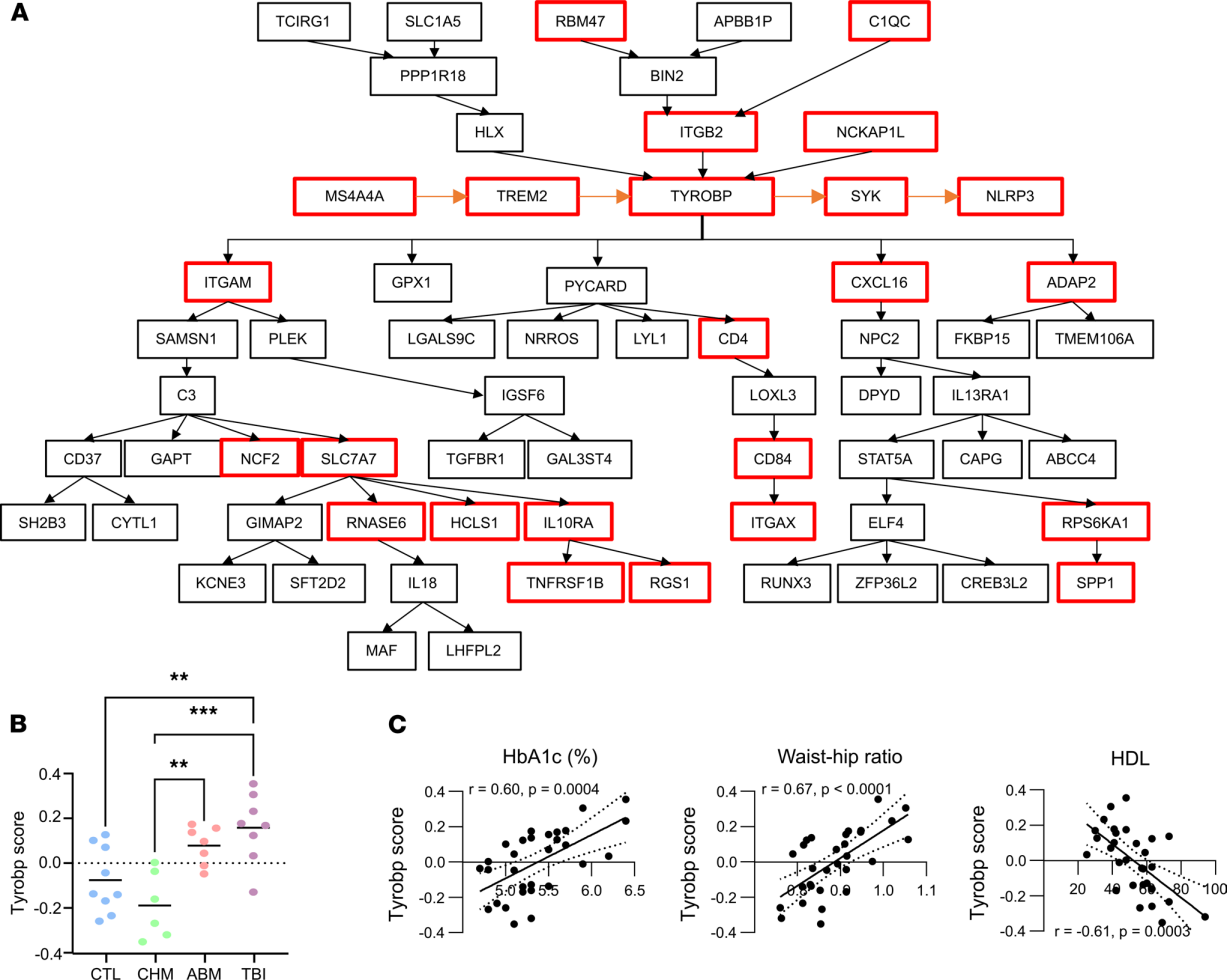
*TREM2-TYROBP* network expression. (**A**) *TYROBP* causal network. The original annotation of this network (nodes and connectivity) is shown in black (43, 74), with red outlines for genes that were upregulated in the TBI and ABM groups. Additional connectivity inferred from the literature (24, 42) is shown in orange. (**B**) Expression score of the 23 genes of the *TREM2-TYROBP* network upregulated in TBI and ABM subjects. **P < 0.01, ***P < 0.001 on 1-way ANOVA with Tukey multitest correction. (**C**) Correlation of *TREM2-TYROBP* network expression scores with HbA1c, WHR, and HDL.

Using the list of DEGs compiled through DESeq2 and WGNCA/QuSAGE, we next conducted pathway analysis using NDex Bio’s integrated query feature (39), which uncovered the *TYROBP* causal network as the top-scoring pathway (Figure 5A), with 19 of 60 pathway genes represented in our gene list (*P* < 1 × 10^–12^). QuSAGE of the C2 canonical pathway gene sets from MSigDB (40) also reveals this network as a top hit (Supplemental Figure 6A). *TYROBP*, also called DAP12, is a transmembrane adaptor that links myeloid surface receptors to downstream signaling effectors (41). The current network annotation does not include a major upstream regulator of *TYROBP*, the transmembrane receptor *TREM2* (24), nor its downstream signaling cascade centered on *SYK* (24), which includes *NLRP3* activation (42) (Figure 5A). Additionally, *MS4A4A* has been shown to regulate cleavage of TREM2 to its soluble form (36). *TREM2*, *SYK*, *NLRP3*, and *MS4A4A* transcripts are all upregulated in the TBI and ABM groups (Supplemental Tables 2 and 7), thus extending representation of the *TYROBP* network to 23 genes. As expected, gene expression scores for the 23-gene *TREM2-TYROBP* signature were enriched in the ABM and TBI groups compared with the CTL and CHM groups (Figure 5B) and were strongly and positively correlated with HbA1c (*r* = 0.60, *P* < 0.001) and WHR (*r* = 0.68, *P* < 0.0001) and negatively correlated with HDL (*r* = –0.58, *P* < 0.001; Figure 5C). ANCOVA examining *TREM2-TYROBP* expression across groups while controlling for age showed a significant group effect (*P* = 0.00063), while age was not significant (*P* = 0.064). Enrichment of select components of the network in the TBI and ABM groups was confirmed by qPCR (Supplemental Figure 6B, *P* = 0.0051 for group effect on rank-transformed mixed effects model; FDR-adjusted *P* = 0.052 for ABM versus CHM; *P* = 0.002 for TBI versus CHM; *P* = 0.007 for TBI versus CTL).

**Figure 6:**
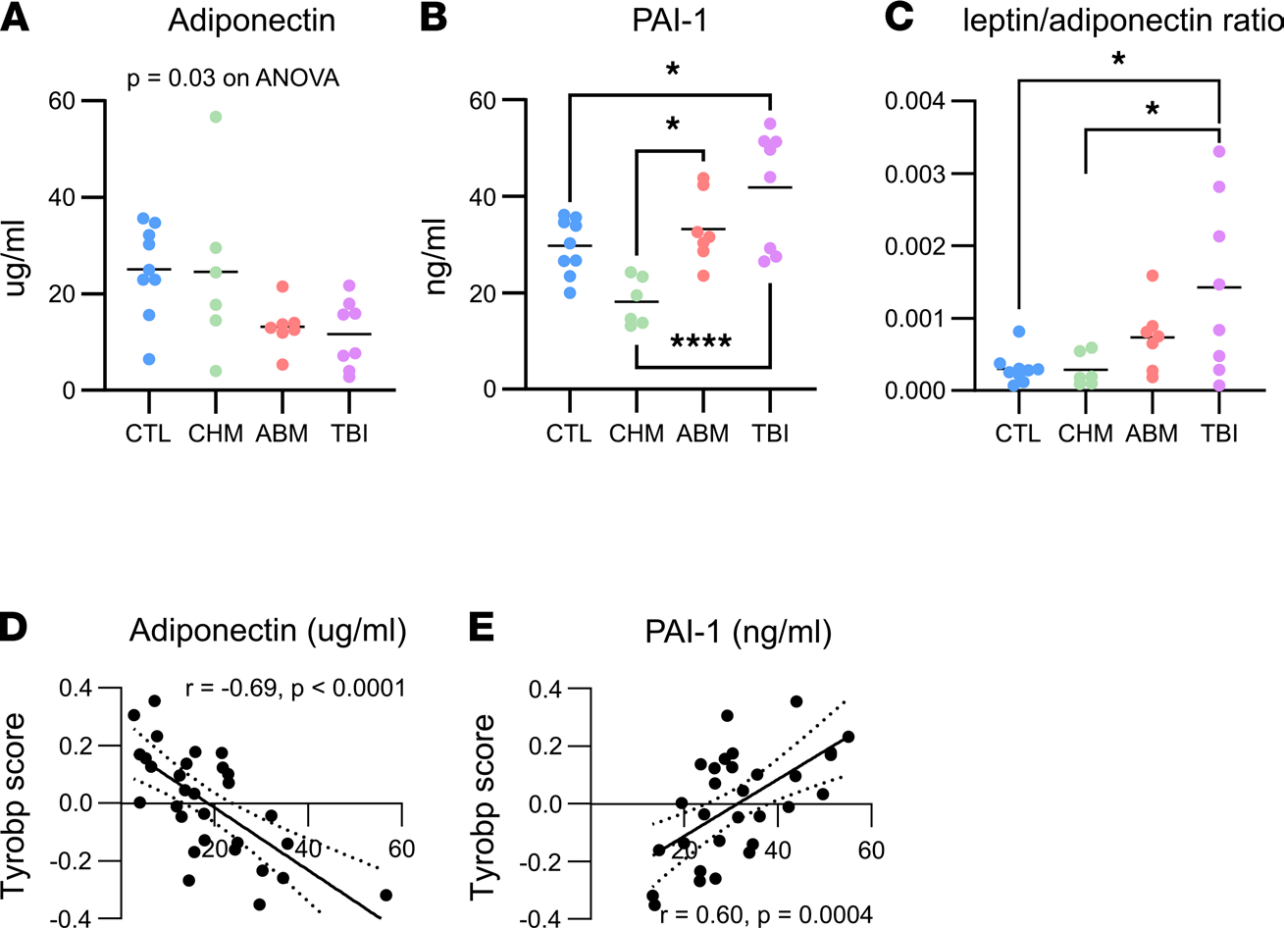
Dysregulated adipokine secretion. (**A** and **B**) Serum adiponectin (**A**) (*P* = 0.03 on 1-way ANOVA; no significant post hoc pairwise comparisons) and PAI-1 levels (**B**) (*P < 0.05, ****P < 0.0001 on one-way ANOVA with Tukey’s multi-test correction). (**C**) Leptin-adiponectin ratio. *P < 0.05 on 1-way ANOVA with Tukey multitest correction for post hoc pairwise comparisons. (**D** and **E**) Correlation of *TREM2-TYROBP* network expression scores with serum adiponectin and PAI-1 levels.

The *TREM2-TYROBP* network was originally described in an analysis of central regulators of gene expression in the brains of patients with Alzheimer’s (43); however, increasing evidence also supports activity of this pathway in SAT, with associated adverse metabolic outcomes. In the DioGenes (44) and METSIM (45) studies of overweight or obese participants, SAT expression of components of the *TREM2-TYROBP* network was found to correlate with clinical measures of insulin resistance (Supplemental Tables 12 and 13). In another study of obese adolescents with MRI-based measurements of VAT and SAT depot sizes, participants with more VAT showed decreased insulin sensitivity and increased macrophage infiltration and expression of NLRP3 inflammasome–related genes in SAT biopsies (46). Overall, *TREM2-TYROBP* network activity in SAT appears to be linked to impaired systemic glucose homeostasis, possibly through disruption of the endocrine function of adipose, which includes secretion of signaling molecules or adipokines that regulate global homeostatic processes such as insulin sensitivity. We profiled a number of known adipokines (Supplemental Figure 6C) and observed a qualitative decrease in serum adiponectin (Figure 6A) and a statistically significant increase in PAI-1 in the ABM and TBI groups (Figure 6B). Although leptin levels were not different among the groups, the leptin/adiponectin ratio, which has been shown to be a predictor of metabolic syndrome (47) and insulin resistance (48), was significantly increased in the TBI group and trending upward in the ABM group (Figure 6C). These changes may explain the early phenotype of insulin resistance in the TBI and ABM groups (Figure 1, F and G), as adiponectin improves insulin sensitivity (49), while PAI-1 promotes insulin resistance (50). As with the clinical metrics associated with worse metabolic health, adiponectin and PAI-1 levels are also correlated with the *TYROBP* network (Figure 6, D and E) and green module (Supplemental Figure 6D) expression in the expected directions.

## Discussion

We report here the first study to our knowledge of the late response of human adipose tissue to therapeutic radiation for childhood cancer. This response constitutes an important mechanistic link between the pathophysiology of RT-induced injury to normal tissue and the clinical observation that many childhood cancer survivors treated with TBI or abdominal RT suffer increased risk of cardiometabolic diseases such as DM (4, 6, 7). Although significant efforts have been made to deintensify childhood cancer treatments to decrease the risk of late effects, there remain sizeable populations of high-risk patients who will require more intensive treatments (51, 52). Understanding the molecular and cellular mechanisms driving development of late effects in this patient population will enable early identification of those at risk and implementation of interventions to prevent progression to debilitating disease.

RT-induced injury to the adipose tissue, a major endocrine organ, has received comparatively little consideration as a mechanism for the development of DM, despite animal data suggesting that RT exposure reduces the proliferative capacity of preadipocytes (12) and leads to adipose tissue macrophage accumulation (14), changes that have been linked to systemic insulin resistance in animal models and human patients (53–55). Our data expand these findings by characterizing a diverse macrophage population in irradiated SAT that is composed of cells with both proinflammatory and tissue-remodeling functions (Figure 4D). This macrophage population is marked by upregulation of the *TYROBP* network (Figure 5A), a damage-response pathway first implicated in the control of amyloid plaques by microglia in Alzheimer’s disease (24). More recent work has uncovered additional roles for this pathway in the clearance of dying adipocytes in obese VAT (56). Although our data do not reveal the triggering signal for *TREM2-TYROBP* activation in irradiated adipose, the persistence of this pathway in the TBI and ABM groups suggests ongoing tissue injury and immune remodeling, with negative consequences for normal adipose function, including altered secretion of adipokines such as adiponectin and PAI-1 that have significant roles in regulating systemic insulin responsiveness. In particular, we observed decreased adiponectin and increased PAI-1 levels in the TBI and ABM groups (Figure 6, A and B), which could explain their emerging phenotype of insulin resistance (Figure 1, F and G) (49, 50). PAI-1 is additionally linked to the development of atherosclerotic plaques and coronary artery thrombosis (57); thus, the increased levels seen in the TBI and ABM groups could explain the increased risk of cardiovascular disease seen in similar cohorts of childhood cancer survivors (4).

Notably, while SAT is thought to secrete the majority of circulating adiponectin (58), PAI-1 is secreted primarily by VAT (59) but is also increased in SAT of obese patients (60). Therefore, the increased circulating levels of PAI-1 in the TBI and ABM groups could indicate expansion of VAT, increased secretion by SAT as it takes on more VAT-like properties, or both. Although not profiled in this study, the VAT of childhood cancer survivors who received TBI or abdominal RT may also become dysfunctional. One hypothesis for the pathogenesis of metabolic syndrome is exhaustion of existing adipose tissue capacity to accommodate caloric excess, leading to ectopic deposition of lipids and subsequent development of insulin resistance (61). As DM can develop in the absence of obesity in childhood cancer survivors (6), an analogous mechanism of adipose dysfunction, albeit from RT instead of caloric excess, could explain the glucose dysregulation observed in survivors treated with abdominal RT or TBI.

Although the inciting events leading to adipose dysfunction are different between childhood cancer survivors and obesity in the general population, our data show that they converge on similar molecular and cellular signatures, including macrophage infiltration (54) and activation of the *TREM2-TYROBP* network (44–46). Intriguingly, the highest BMI of our study cohort occurred in the CTL group, and this participant consistently behaved more similarly to the TBI and ABM groups compared with other CTL or CHM participants, including on scores of green module and *TREM2-TYROBP* network expression, although the macrophage cell fraction was not elevated. Given the cross-sectional nature of our study with sampling from only 1 time point fairly distant (>20 years) from completion of RT, we cannot rule out events occurring early after treatment that would be specific to RT-induced injury and only later converge on the more common pathways of adipose dysfunction that lead to insulin resistance. These early processes may include radiation-induced tissue hypoxia (62) and extracellular matrix remodeling (63), both of which have also been implicated in the pathogenesis of adipose tissue dysfunction (64).

Our study has a number of limitations, primarily a small and heterogeneous population, which may limit the generalizability of the findings. In general, we found larger effect sizes in the TBI group compared with the ABM group, and this could be a result of the uncertainty of sampling irradiated adipose in the ABM group or of the physiologic action of other tissues that are irradiated in TBI, such as the hypothalamic-pituitary axis or pancreas, on adipose function and overall metabolic health. Finally, RT treatment techniques have changed significantly within the past 10–15 years, with increasing usage of conformal fields and proton therapy, possibly limiting the applicability of our findings to future cohorts of survivors. However, adipose appears to be a radiosensitive tissue, based on prior animal studies (12) and our data showing profound and chronic gene expression changes even after receipt of the low doses used in TBI. Current RT-planning practice does not give special consideration for minimizing RT dose to adipose; therefore, our findings will likely remain clinically relevant.

The goal of this study was to identify molecular changes in irradiated abdominal adipose to serve as foundational data for hypothesis generation and future validation in prospective cohorts. Future work profiling earlier changes in irradiated adipose can uncover the pathogenic mechanisms that lead to the molecular changes we observed. Orthogonal studies such as MRI or CT determination of individual adipose depot sizes and core biopsies to determine the tissue architecture of irradiated adipose will enable better understanding of changes in adipose biology at different scales. Finally, mechanistic studies using mouse models can confirm the specific molecular pathways that link RT adipose injury to the development of cardiometabolic disease, with the eventual goal of targeting these pathways therapeutically to alter the disease course.

## Methods

### Subject recruitment and procedures.

Patients followed in the MSKCC Long-Term Follow-Up Clinic were screened for eligibility prior to scheduled clinic visits. Adult (≥18 years of age) survivors of childhood cancer who had completed treatment 2 or more years prior to enrollment were eligible. Treatment consisted of either exposure to abdominal RT (ABM, *n* = 51) or TBI (*n* = 64) in conjunction with chemotherapy, or chemotherapy alone with no RT exposure (CHM, *n* = 45). RT to other sites was allowed in the ABM and TBI groups. A control group (CTL) of healthy volunteers (*n* = 16) was recruited by the Rockefeller University Hospital. Exclusion criteria (designated “medical contraindication” in Figure 1A) included BMI ≥ 30 kg/m^2^; diagnosis of DM; pregnancy; current use of adipose-altering medications (insulin, thiazolidinediones, atypical antipsychotics, topiramate, or stimulants), anticoagulants, NSAIDs, or aspirin; HbA1c > 6.5%; platelet count, PT, and PTT outside normal limits; and known symptomatic coronary artery disease. Subjects were also excluded if they had inadequate SAT for biopsy as determined on physical exam. Power calculations determined that 40 subjects (10 per group) were needed to provide 80% power at 5% significance to detect moderate-to-large effect sizes in ANOVA models, assuming that the average coefficient of variation was 70%. The protocol was opened in June 2017, and the last subject was enrolled in January 2020 before the principal investigators decided to proceed with data analysis, given slow accrual. At that point, 35 subjects had enrolled.

During a single clinical visit after an overnight fast, participants underwent venipuncture to obtain blood samples for complete blood count (CBC), prothrombin time/partial thromboplastin time (PT/PTT), lipid panel, HbA1c, fasting glucose, and high sensitivity C-reactive protein (hsCRP). Additional serum and plasma samples were banked for later investigational testing. Body composition (percent lean and fat mass) was measured using a BodPod air plethysmograph (COSMED); 2 CTL subjects did not complete this measurement due to equipment malfunction and were excluded from analyses of body fat and lean mass composition. Biopsy of the abdominal SAT was performed under local anesthesia through a small incision in the lower abdomen, followed by aspiration of 2–3 g of tissue with a 4 mm blunt-end liposuction needle. Patients were discharged after biopsy and monitored by telephone for postprocedure complications, which were minor (primarily bruising, discomfort) and resolved without sequelae.

### RNA isolation and sequencing.

RNA was extracted from frozen adipose tissue with Trizol (Invitrogen) and purified with RNAeasy columns (Qiagen). RNA integrity number (RIN) was measured with a bioanalyzer (Agilent). Library preparation was performed by the Rockefeller Genomics Resource Center using 100 ng of total RNA and the TruSeq Stranded Total RNA Library Prep Kit with Ribo-Zero Human/Mouse/Rat (Illumina). Libraries were prepared with unique barcodes and pooled at equal molar ratios. The pool was denatured and sequenced on an Illumina NovaSeq 6000 sequencer using v.1.5 reagents, an S1 flow cell, and NovaSeq Control Software v.1.7.0 to generate 50 bp paired-end reads, following the manufacturer’s protocol. The sequencing data for 27 of the 30 participants are available from GEO, accession GSE184148; 2 CHM and 1 CTL participants declined to have their data shared publicly.

### RNA-seq data processing and differential expression analysis.

Sequencing reads were aligned and quantified using Salmon (v.0.8.2) (65). Differential gene analysis was performed using DESeq2 (17) (R package v.1.20.0) with default negative binomial modeling and Wald’s test with a significance cut-off of adjusted *P* < 0.05. For all downstream applications (WGCNA, QuSAGE, cell type deconvolution) requiring normalized read counts, results from the variance stabilized transformation (VST) algorithm implemented in DESeq2 were used. The R packages volcano3D (18) and EnhancedVolcano (66) were used to plot 3-way and pairwise gene expression comparisons, respectively. Pairwise volcano plots were generated using direct output from DESeq2. For 3-way comparisons, the likelihood ratio test was used as the statistical test for between-group difference.

### GO analysis.

GO enrichment was performed with topGO (67) (v.2.42.0) using Fisher’s exact test. Enrichment scores are taken to be –log_10_(raw *P* value) of each term.

### WGNCA and QuSAGE.

The WGCNA (19) R package (v.1.70.3) was used to construct modules of correlated gene expression in a blockwise manner to reduce computation time. Correlation was calculated using the “bicor” algorithm implemented in the package and a soft thresholding power of 24 (empirically determined) to reach scale-free topology. The module eigengenes calculated by the package function “moduleEigengenes” were used as module expression scores where indicated, and this function was adapted to calculate aggregate expression for other groups of genes such as those comprising the *TREM2-TYROBP* network. QuSAGE (20) (R package v.2.24.0) was performed using the WGNCA modules as gene sets or the C2 canonical pathways from MSigDB (40) and compared the ABM and TBI subjects together (RT group) against the CTL and CHM subjects together (non-RT group). Individual gene enrichment scores or “activities” in QuSAGE are calculated as mean fold changes between comparison groups. Top genes are considered as those whose lower bound of the 95% CI around the gene enrichment score is greater than the mean enrichment score of the entire module, which is the pathway enrichment score.

### Cell type deconvolution.

The normalized count matrix from the scRNA experiment published in Vijay et. al (25) was downloaded from GEO (accession number GSE136230). Seurat v.4.0.1 (68) was used to cluster the subset of cells isolated from SAT, using the first 25 principal components and a resolution of 0.75. Cluster markers were obtained from the “FindAllMarkers” function with a requirement of marker expression in at least 25% of cells in a cluster. Cluster identities were verified against identities published in Vijay et al. (25) and automated annotation using SingleR (v.1.4.1) (69) and the Encode and Human Primary Cell Atlas databases (70–72). Comparisons of the Vijay clusters (25) and our reclustering results are shown in Supplemental Table 8. The cluster-annotated normalized count matrix was forwarded to CIBERSORTx (26) to calculate an adipose tissue signature matrix. The bulk RNA-seq data (VST-normalized) was then deconvoluted against this matrix using the recommended S-mode batch correction. The resulting cell fraction data were modeled using the aligned rank transformation method for repeated measures with interaction (73), which was appropriate given that the fractions sum to unity and are thus not entirely independent. FDR-adjusted *P* values for post hoc pairwise comparisons were calculated using estimated marginal means and are listed in Supplemental Table 9. The FDR was calculated using only group comparisons within each cell type, instead of possible pairwise comparisons within the data.

### Macrophage subcluster deconvolution.

The cells from clusters *m1* and *m2* were subsetted from the larger data set and clustered at resolution 0.35 using the first 15 principal components, which resulted in 7 clusters. Cluster markers were obtained from the “FindAllMarkers” function with a requirement of marker expression in at least 25% of cells in a cluster; these are listed in Supplemental Table 10. Repeated clustering at resolution 1.0 resulted in 12 clusters, which did not result in separation of cluster *M-A* markers (Supplemental Figure 5C) into different clusters (Supplemental Figure 5D), suggesting that using resolution of 0.35 did not result in underclustering. Cell type annotation of the single-cell count matrix was updated with the macrophage subcluster identities *M-A* to *M-G* in place of *m1* and *m2* identities. CIBERSORTx was repeated with this annotation with the same settings as above. The resulting macrophage fractions were plotted and tested for differences among the groups using the Kruskal-Wallis test, followed by the Dunn test for post hoc comparisons with FDR adjustment. Results are listed in Supplemental Table 11. The Kruskal-Wallis test was selected, as the macrophage fractions did not follow a normal distribution. Aligned rank transformation was not used, given that multiple macrophage clusters had no representation in a large number of samples, resulting in a large number of ties and increased type 1 errors for the aligned rank method.

### qPCR.

cDNA was synthesized from 1 μg of RNA using the High Capacity cDNA Reverse Transcription Kit (Applied Biosciences). Of note, 2 samples (1 from ABM group, 1 from CHM group) did not have sufficient RNA remaining after sequencing to generate cDNA for qPCR, resulting in 28 samples available for analysis. qPCR was performed using Power SYBR Green (Invitrogen) in 384-well plate format on the QuantStudio 6 Flex Real-Time PCR System (Thermo Fisher Scientific). Relative gene expression difference was calculated using the ΔΔCT method normalizing to *EEF2*. Relative expression values of all transcripts reported in Supplemental Figure 4B or Supplemental Figure 6B were modeled as linear mixed effects models with individual samples as a random effect and subject group as a fixed effect. Due to the nonnormal distribution and heteroscedasticity of the data, a rank transformation was performed prior to running the model. Post hoc pairwise comparisons between groups were performed FDR correction on Tukey contrasts.

### Serum adipokine analysis.

Subject serum samples were sent to Eve Technologies for analysis on their Human Metabolic Hormone Array 9-Plex (HDMET9) and Human Adipokine Array 5-plex (HDADK5) assays to profile leptin, adipsin, resistin, lipocalin-2, adiponectin, and PAI-1 levels.

### Statistics.

Analyses were performed in R or GraphPad Prism. Unless otherwise described in the main text or the relevant section of the Methods, 1-way ANOVA with Tukey multitest correction for post hoc pairwise comparisons was used to determine differences among treatment groups. Two-tailed Student’s t test was used for comparison when only 1 groups were relevant (e.g., RT dose in TBI and ABM groups). One-way ANCOVA was performed using type III sum of squares. The underlying statistical models of DESeq2, WGCNA, QuSAGE, topGO, and Seurat were used with default settings unless otherwise indicated. *P* values less than 0.05 were considered significant.

### Study approval.

Written informed consent was obtained from all participants prior to enrollment in the study. This study and all relevant procedures were reviewed and approved by the IRB of both MSKCC (protocol no. 17-314) and The Rockefeller University (protocol no. PCO-0902).

## Author contributions

DNF and PC conceived the study. DNF screened and recruited study participants. JMW performed the abdominal adipose biopsies. XH and OAM performed in vitro experiments. XH analyzed the data and performed bioinformatic and statistical analyses. TSC and JDL assisted with bioinformatic analyses. CSJ provided statistical analyses. ET provided intellectual input. XH and PC wrote the manuscript with input from all authors.

## Supplementary Material

Supplemental data

ICMJE disclosure forms

Supplemental tables 1-13

## Figures and Tables

**Table 1:**
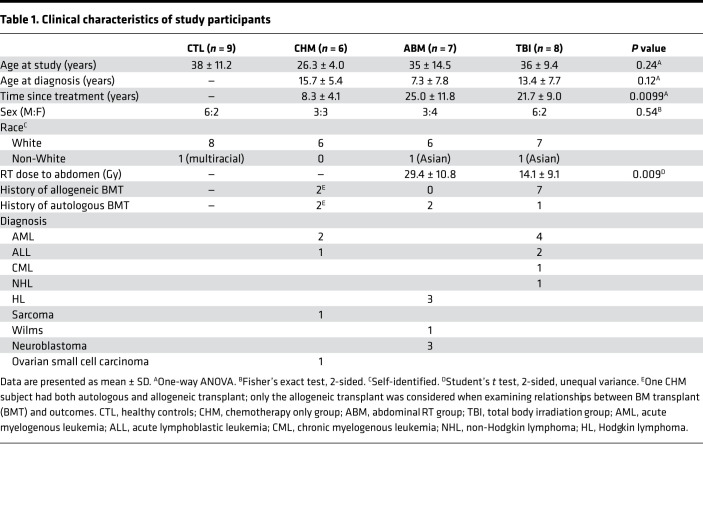
Clinical characteristics of study participants

## Version 1 09/23/2021

In-Press Preview

## Version 2 11/08/2021

Electronic publication
