# Supplementary material for: Therapeutic radiation exposure of the abdomen during childhood induces chronic adipose tissue dysfunction
Source: JCI Insight. 2021 Nov 8;6(21):e153586. doi: 10.1172/jci.insight.153586 (PMC8663557; doi:10.1172/jci.insight.153586)
Supplement: Supplemental data [file jciinsight-6-153586-s183.pdf]

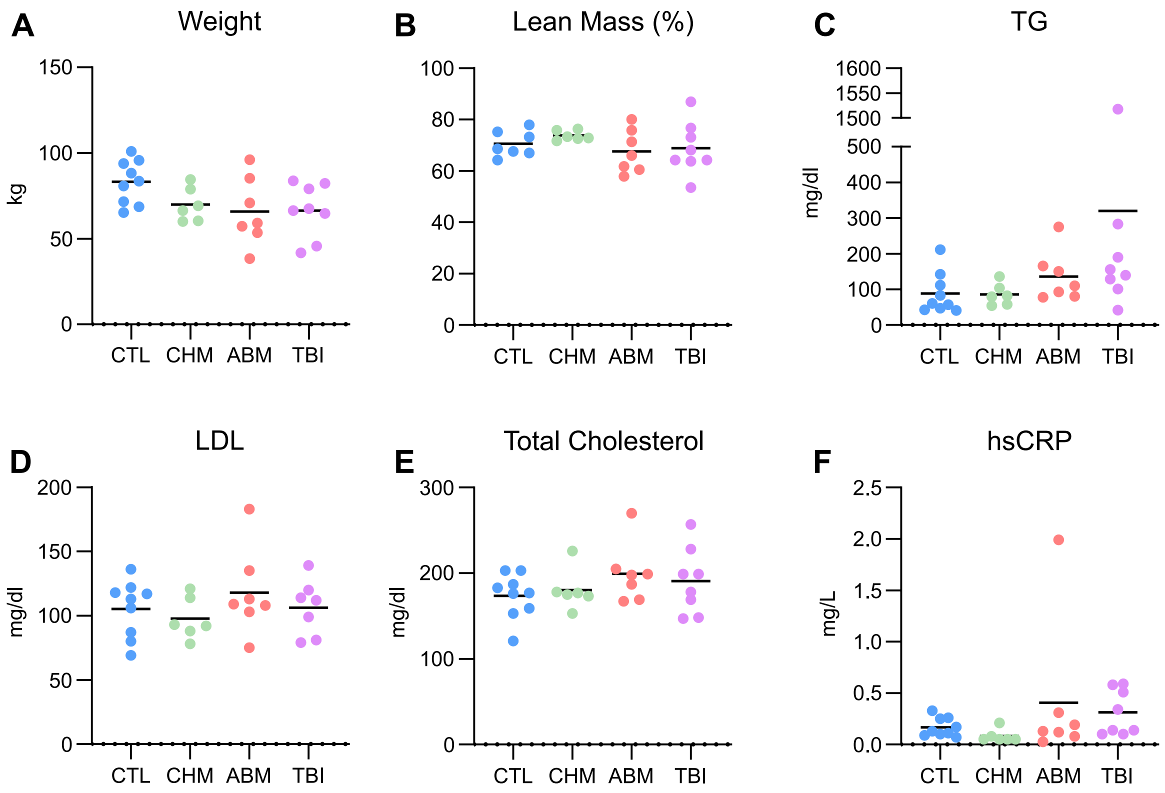

Supplemental Figure 1: Additional clinical characteristics of study participants. (**A**) Weight, (**B**) lean mass %, (**C**) triglycerides (TG), (**D**) LDL, (**E**) total cholesterol, and (**F**) high-sensitivity C-reactive protein (hsCRP) measurements. No comparisons were significant on one-way ANOVA. One TBI subject did not have an LDL measurement due to extremely high TG.

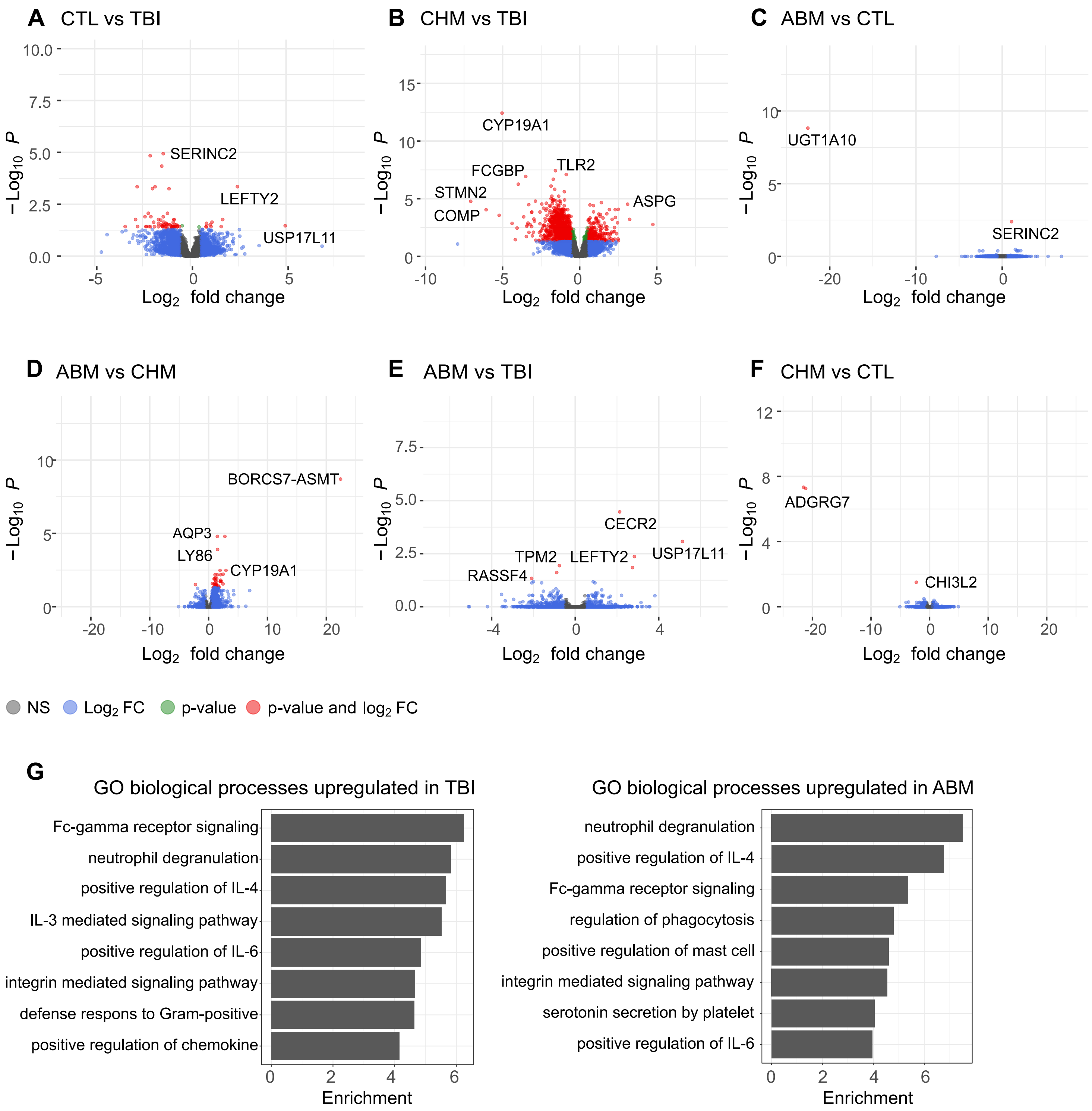

Supplemental Figure 2: Differential gene expression analysis. (**A**)-(**F**) Volcano plots for pairwise comparisons indicated. Genes with p-value < 0.05 on Wald's test implemented in DESeq2 and/or log2 fold-change >0.5 are highlighted. (**G**) Gene ontology (GO) enrichment analysis of genes upregulated in either TBI or ABM groups, scored as -log10 of the adjusted p-value from topGO.

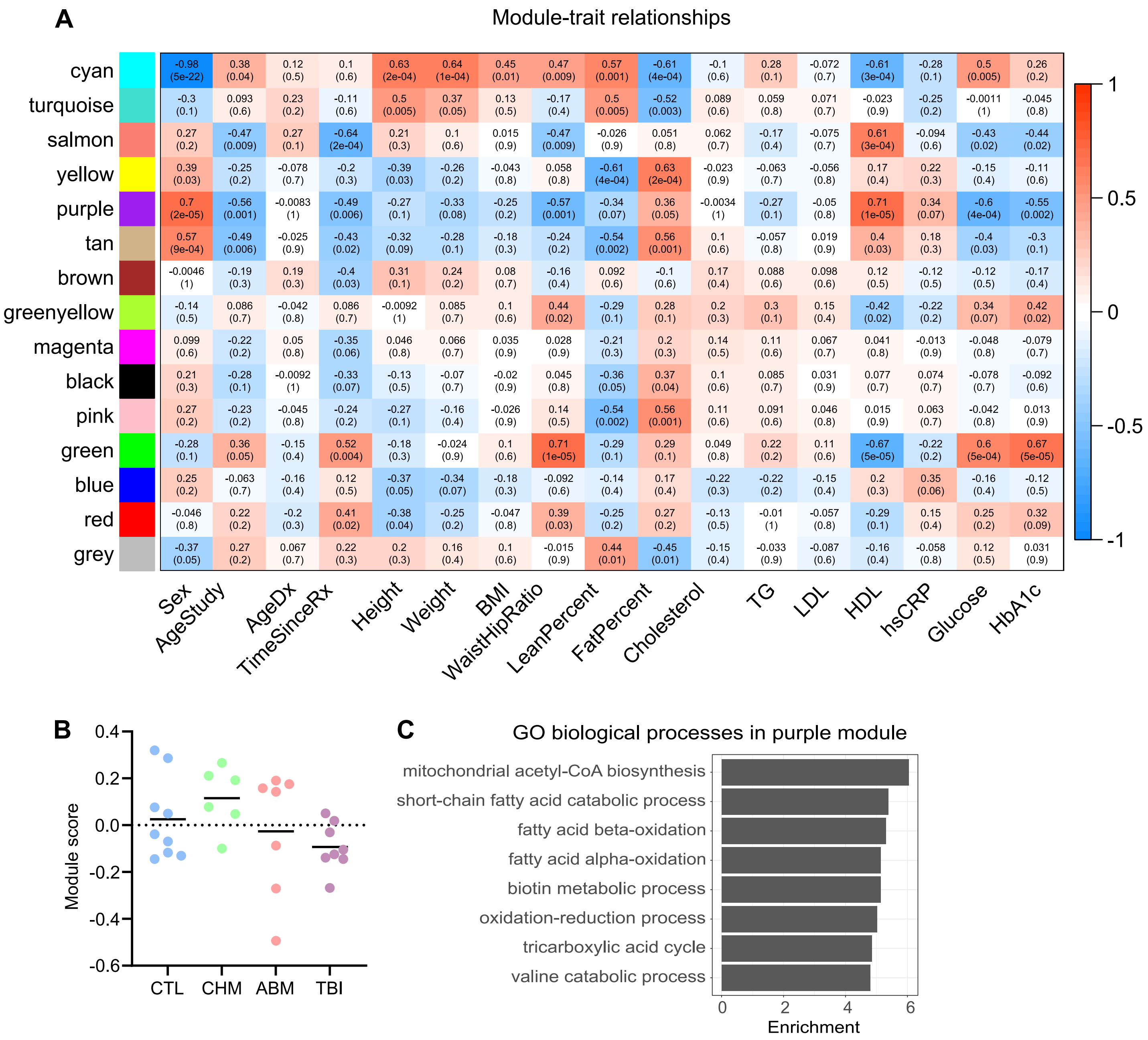

Supplemental Figure 3: WGCNA modules. (**A**) Correlation matrix between WGCNA modules (colors on y-axis) and participant clinical traits (x-axis). Correlation coefficients are shown with associated p-values in parentheses. AgeStudy: age at study. AgeDx: age at diagnosis. TimeSinceRx: time since treatment. BMI: body mass index. TG: triglycerides. LDL: low density lipoprotein. HDL: high density lipoprotein. hsCRP: high sensitivity C-reactive protein. HbA1c: glycated hemoglobin. (**B**) Purple module scores calculated from WGCNA's eigengene function. No significant comparisons on one-way ANOVA. (**C**) Gene ontology (GO) enrichment analysis of purple module genes, scored as -log10 of the adjusted p-value from topGO.

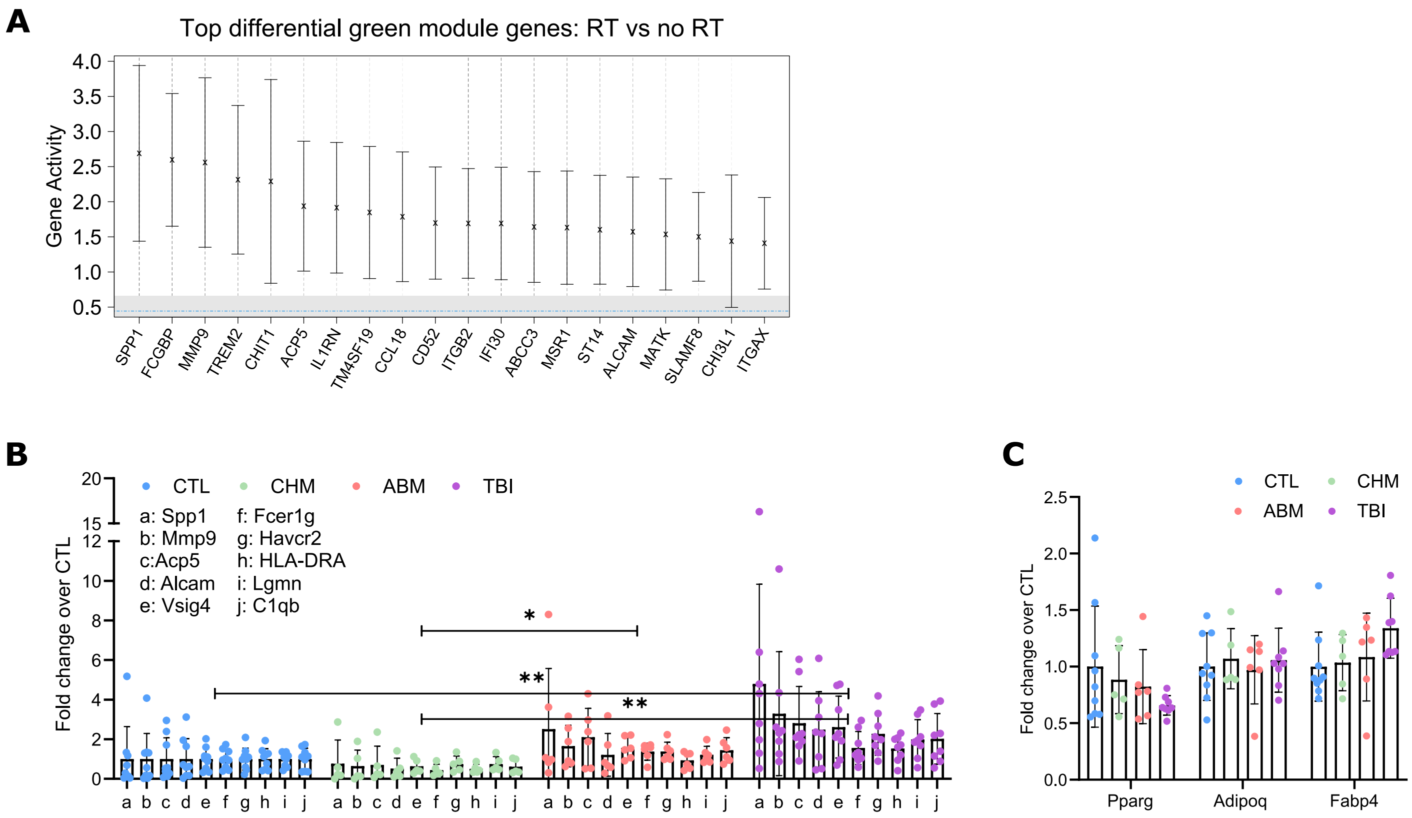

Supplemental Figure 4: Significant module genes. (**A**) Top twenty green module genes by QuSAGE activity score (mean fold-change of expression) in RT vs no RT comparison. Error bars depict 95% confidence interval of activity score estimate, calculated by QuSAGE. (**B**) Transcript levels by qPCR of top-scoring QuSAGE genes *(SPP1, MMP9, ACP5, ALCAM, VSIG4)*, DESeq2-identified differentially expressed genes *(FCER1G, HAVCR2, HLA-DRA, LGMN)*, and (**C**) adipogenesis genes *(PPARG, ADIPOQ, FABP4).* *p < 0.05, **p < 0.01 on post-hoc pairwise comparisons between groups with FDR adjustment after ANOVA on a linear mixed effects model with rank transformation. The adipogenesis genes were not different among groups on Kruskal-Wallis test. Of note, two subjects (one from ABM group, one from CHM group) did not have sufficient RNA remaining after sequencing to generate cDNA for qPCR, resulting in 28 samples available for analysis. Error bars depict standard deviation.

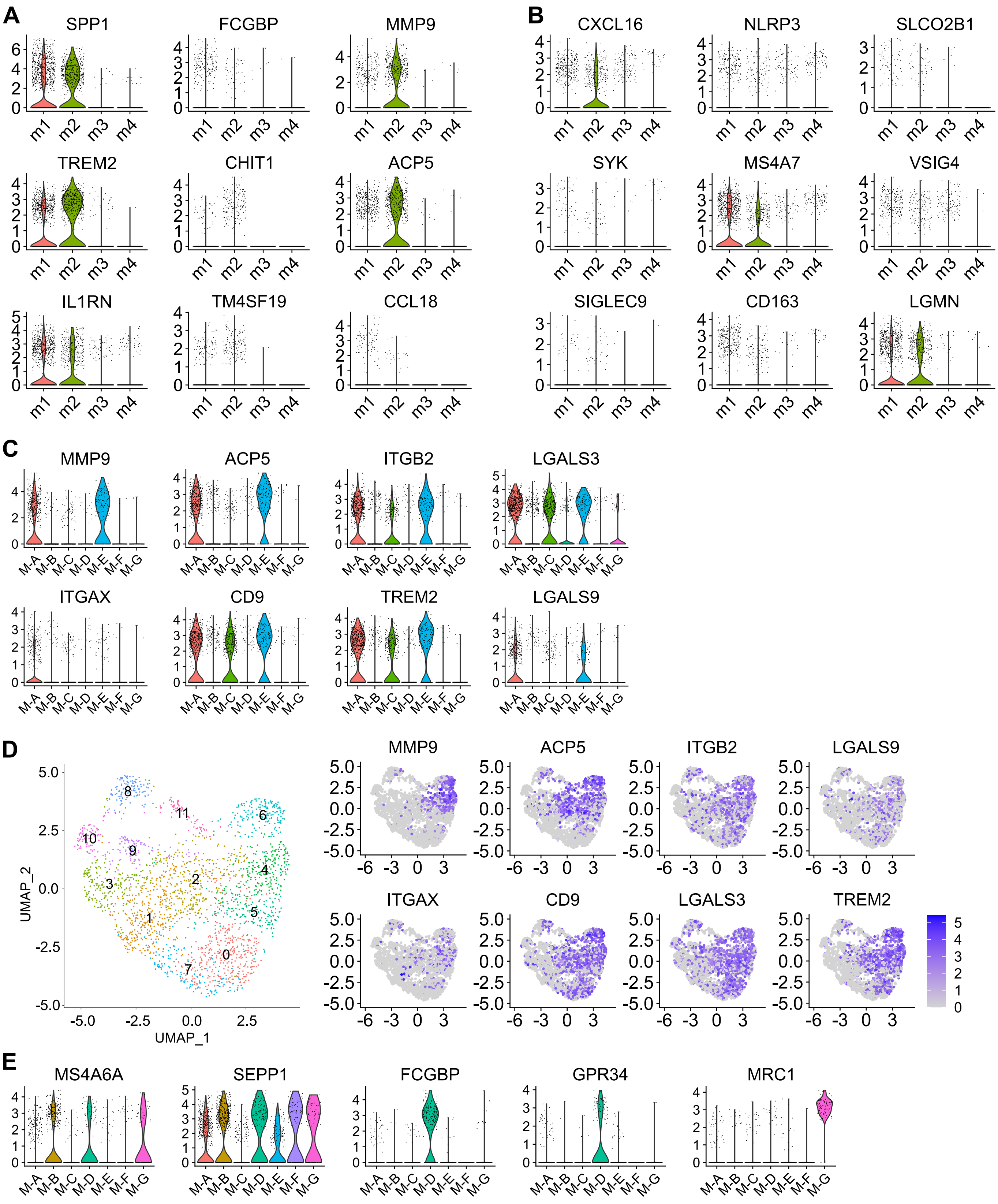

Supplemental Figure 5: Macrophage clusters in irradiated adipose tissue. Violin plots of (**A**) QuSAGE- and (**B**) DESeq2-identified differentially expressed genes among myeloid clusters identified from Vijay et. al SAT scRNA data. (**C**) Violin plots of indicated markers in macrophage subclusters. (**D**) Re-clustering of macrophage cells at higher resolution into 12 groups; distribution of same markers in (C) across these clusters. Color scale depicts expression levels. (**E**) Violin plots of indicated markers in macrophage subclusters.

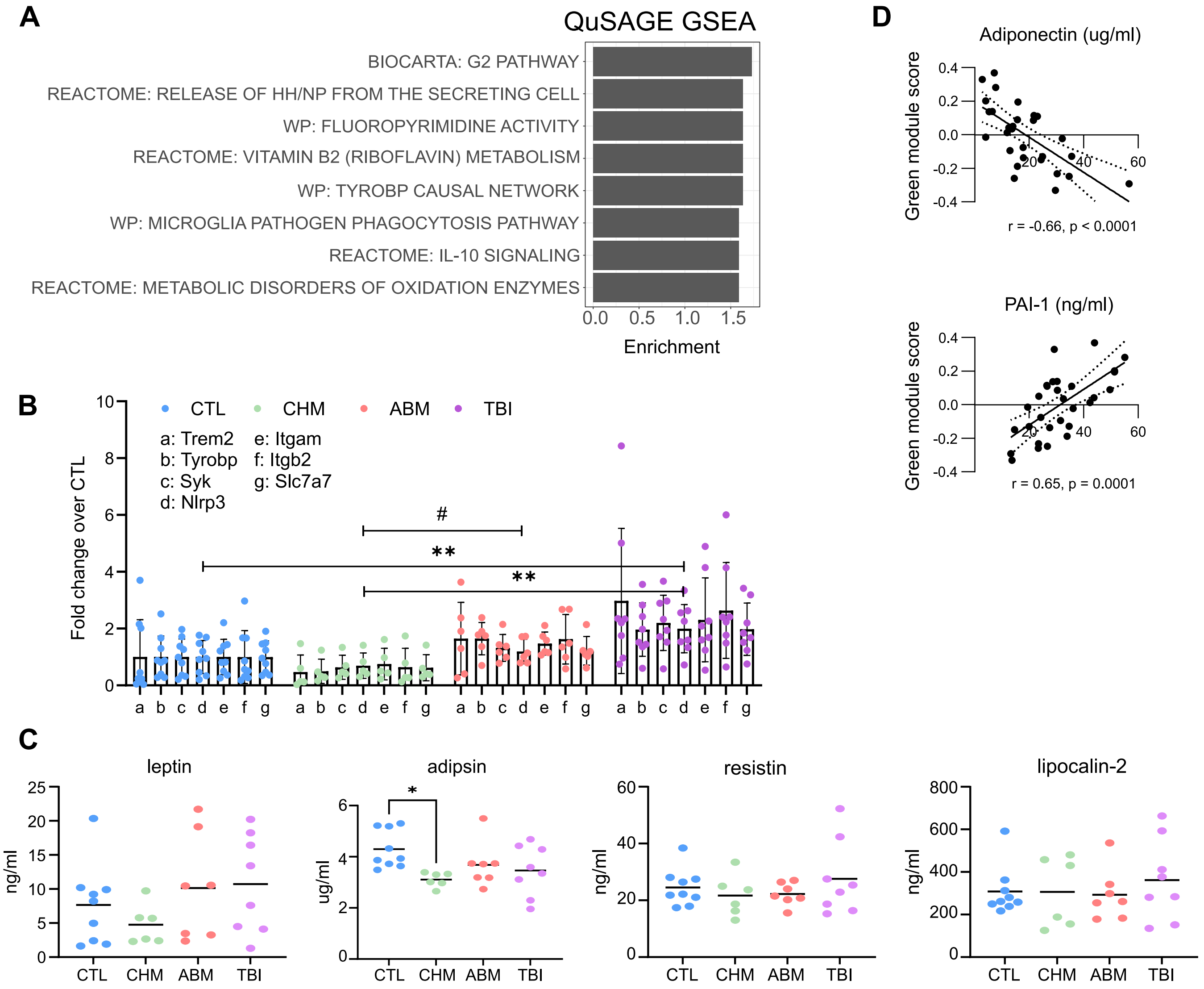

Supplemental Figure 6: TYROBP network and dysregulated adipokine secretion. (**A**) QuSAGE of C2 canonical pathways from MSigDB. WP: WikiPathways. Enrichment is the -$\log_{10}$ of the QuSAGE pathway activity score. (**B**) Transcript levels by qPCR of select genes of *TREM2-TYROBP* network upregulated in TBI and ABM subjects. #p = 0.052, **p < 0.01 on post-hoc pairwise comparisons with FDR adjustment after ANOVA on a linear mixed effects model with rank transformation. Error bars depict standard deviation. Of note, two subjects (one from ABM group, one from CHM group) did not have sufficient RNA remaining after sequencing to generate cDNA for qPCR, resulting in 28 samples available for analysis. (**C**) Serum levels of indicated adipokines. *p < 0.05 on one-way ANOVA with Tukey multi-test correction for post-hoc pairwise comparisons. (**D**) Correlation of green module expression scores with adiponectin and PAI-1 levels.
