# Supplementary material for: Therapeutic radiation exposure of the abdomen during childhood induces chronic adipose tissue dysfunction
Source: JCI Insight. 2021 Nov 8;6(21):e153586. doi: 10.1172/jci.insight.153586 (PMC8663557; doi:10.1172/jci.insight.153586)
Supplement: ICMJE disclosure forms [file jciinsight-6-153586-s184.pdf]

ICMJE DISCLOSURE FORM

Date:__9/1/21____
Your Name:_Xiaojing Huang____
Manuscript Title: Therapeutic abdominal radiation during childhood induces chronic adipose tissue dysfunction
Manuscript number (if known): 153586-JCI-CMED-PRESUB

In the interest of transparency, we ask you to disclose all relationships/activities/interests listed below that are related to the content of your manuscript. "Related" means any relation with for-profit or not-for-profit third parties whose interests may be affected by the content of the manuscript. Disclosure represents a commitment to transparency and does not necessarily indicate a bias. If you are in doubt about whether to list a relationship/activity/interest, it is preferable that you do so.

The following questions apply to the author's relationships/activities/interests as they relate to the current manuscript only.

The author's relationships/activities/interests should be defined broadly. For example, if your manuscript pertains to the epidemiology of hypertension, you should declare all relationships with manufacturers of antihypertensive medication, even if that medication is not mentioned in the manuscript.

In item #1 below, report all support for the work reported in this manuscript without time limit. For all other items, the time frame for disclosure is the past 36 months.

		**Name all entities with whom you have this relationship or indicate none (add rows as needed)**	**Specifications/Comments (e.g., if payments were made to you or to your institution)**
		**Time frame: Since the initial planning of the work**	
1	All support for the present manuscript (e.g., funding, provision of study materials, medical writing, article processing charges, etc.) **No time limit for this item.**	____ None<br>Rockefeller University Hospital	Pilot grant
		National Institute of General Medical Sciences	T32GM007739
		National Center for Advancing Translational Sciences	UL1TR001866
		National Cancer Institute	P30CA008748
		American Cancer Society	133831-CSDG-19-117-01-CPHPS
		American Diabetes Association	1-17-ACE-17
		private donor (Memorial Sloan Kettering Cancer Center	
		**Time frame: past 36 months**	
2		_x___ None	

	Grants or contracts from any entity (if not indicated in item #1 above).		
3	Royalties or licenses	_x___ None	
4	Consulting fees	_x___ None	
5	Payment or honoraria for lectures, presentations, speakers bureaus, manuscript writing or educational events	_x___ None	
6	Payment for expert testimony	_x___ None	
7	Support for attending meetings and/or travel	_x___ None	
8	Patents planned, issued or pending	_x___ None	
9	Participation on a Data Safety Monitoring Board or Advisory Board	_x___ None	
10	Leadership or fiduciary role in other board, society, committee or advocacy group, paid or unpaid	_x___ None	
11	Stock or stock options	_x___ None	
12	Receipt of equipment, materials, drugs, medical writing, gifts or other services	_x___ None	
13	Other financial or non-financial interests	_x___ None	

Please place an "X" next to the following statement to indicate your agreement:

_x_ I certify that I have answered every question and have not altered the wording of any of the questions on this form.

ICMJE DISCLOSURE FORM

Date:__9/1/21____
Your Name:_Olivia Maguire____
Manuscript Title: Therapeutic abdominal radiation during childhood induces chronic adipose tissue dysfunction
Manuscript number (if known): 153586-JCI-CMED-PRESUB

In the interest of transparency, we ask you to disclose all relationships/activities/interests listed below that are related to the content of your manuscript. "Related" means any relation with for-profit or not-for-profit third parties whose interests may be affected by the content of the manuscript. Disclosure represents a commitment to transparency and does not necessarily indicate a bias. If you are in doubt about whether to list a relationship/activity/interest, it is preferable that you do so.

The following questions apply to the author's relationships/activities/interests as they relate to the current manuscript only.

The author's relationships/activities/interests should be defined broadly. For example, if your manuscript pertains to the epidemiology of hypertension, you should declare all relationships with manufacturers of antihypertensive medication, even if that medication is not mentioned in the manuscript.

In item #1 below, report all support for the work reported in this manuscript without time limit. For all other items, the time frame for disclosure is the past 36 months.

		**Name all entities with whom you have this relationship or indicate none (add rows as needed)**	**Specifications/Comments (e.g., if payments were made to you or to your institution)**
**Time frame: Since the initial planning of the work**			
1	All support for the present manuscript (e.g., funding, provision of study materials, medical writing, article processing charges, etc.) **No time limit for this item.**	____ None<br>Rockefeller University Hospital	Pilot grant
		National Institute of General Medical Sciences	T32GM007739
		National Center for Advancing Translational Sciences	UL1TR001866
		National Cancer Institute	P30CA008748
		American Cancer Society	133831-CSDG-19-117-01-CPHPS
		American Diabetes Association	1-17-ACE-17
		private donor (Memorial Sloan Kettering Cancer Center	
**Time frame: past 36 months**			
2		_x___ None	

	Grants or contracts from any entity (if not indicated in item #1 above).		
3	Royalties or licenses	_x___ None	
4	Consulting fees	_x___ None	
5	Payment or honoraria for lectures, presentations, speakers bureaus, manuscript writing or educational events	_x___ None	
6	Payment for expert testimony	_x___ None	
7	Support for attending meetings and/or travel	_x___ None	
8	Patents planned, issued or pending	_x___ None	
9	Participation on a Data Safety Monitoring Board or Advisory Board	_x___ None	
10	Leadership or fiduciary role in other board, society, committee or advocacy group, paid or unpaid	_x___ None	
11	Stock or stock options	_x___ None	
12	Receipt of equipment, materials, drugs, medical writing, gifts or other services	_x___ None	
13	Other financial or non-financial interests	_x___ None	

Please place an "X" next to the following statement to indicate your agreement:

_x_ I certify that I have answered every question and have not altered the wording of any of the questions on this form.

ICMJE DISCLOSURE FORM

Date:__9/1/21____
Your Name:_Jeanne Walker____
Manuscript Title: Therapeutic abdominal radiation during childhood induces chronic adipose tissue dysfunction
Manuscript number (if known): 153586-JCI-CMED-PRESUB

In the interest of transparency, we ask you to disclose all relationships/activities/interests listed below that are related to the content of your manuscript. "Related" means any relation with for-profit or not-for-profit third parties whose interests may be affected by the content of the manuscript. Disclosure represents a commitment to transparency and does not necessarily indicate a bias. If you are in doubt about whether to list a relationship/activity/interest, it is preferable that you do so.

The following questions apply to the author's relationships/activities/interests as they relate to the current manuscript only.

The author's relationships/activities/interests should be defined broadly. For example, if your manuscript pertains to the epidemiology of hypertension, you should declare all relationships with manufacturers of antihypertensive medication, even if that medication is not mentioned in the manuscript.

In item #1 below, report all support for the work reported in this manuscript without time limit. For all other items, the time frame for disclosure is the past 36 months.

		**Name all entities with whom you have this relationship or indicate none (add rows as needed)**	**Specifications/Comments (e.g., if payments were made to you or to your institution)**
		**Time frame: Since the initial planning of the work**	
1	All support for the present manuscript (e.g., funding, provision of study materials, medical writing, article processing charges, etc.) **No time limit for this item.**	____ None<br>Rockefeller University Hospital	Pilot grant
		National Institute of General Medical Sciences	T32GM007739
		National Center for Advancing Translational Sciences	UL1TR001866
		National Cancer Institute	P30CA008748
		American Cancer Society	133831-CSDG-19-117-01-CPHPS
		American Diabetes Association	1-17-ACE-17
		private donor (Memorial Sloan Kettering Cancer Center	
		**Time frame: past 36 months**	
2		_x___ None	

	Grants or contracts from any entity (if not indicated in item #1 above).		
3	Royalties or licenses	_x___ None	
4	Consulting fees	_x___ None	
5	Payment or honoraria for lectures, presentations, speakers bureaus, manuscript writing or educational events	_x___ None	
6	Payment for expert testimony	_x___ None	
7	Support for attending meetings and/or travel	_x___ None	
8	Patents planned, issued or pending	_x___ None	
9	Participation on a Data Safety Monitoring Board or Advisory Board	_x___ None	
10	Leadership or fiduciary role in other board, society, committee or advocacy group, paid or unpaid	_x___ None	
11	Stock or stock options	_x___ None	
12	Receipt of equipment, materials, drugs, medical writing, gifts or other services	_x___ None	
13	Other financial or non-financial interests	_x___ None	

Please place an “X” next to the following statement to indicate your agreement:

_x_ I certify that I have answered every question and have not altered the wording of any of the questions on this form.

ICMJE DISCLOSURE FORM

Date:__9/1/21____
Your Name:_Caroline Jiang____
Manuscript Title: Therapeutic abdominal radiation during childhood induces chronic adipose tissue dysfunction
Manuscript number (if known): 153586-JCI-CMED-PRESUB

In the interest of transparency, we ask you to disclose all relationships/activities/interests listed below that are related to the content of your manuscript. "Related" means any relation with for-profit or not-for-profit third parties whose interests may be affected by the content of the manuscript. Disclosure represents a commitment to transparency and does not necessarily indicate a bias. If you are in doubt about whether to list a relationship/activity/interest, it is preferable that you do so.

The following questions apply to the author's relationships/activities/interests as they relate to the current manuscript only.

The author's relationships/activities/interests should be defined broadly. For example, if your manuscript pertains to the epidemiology of hypertension, you should declare all relationships with manufacturers of antihypertensive medication, even if that medication is not mentioned in the manuscript.

In item #1 below, report all support for the work reported in this manuscript without time limit. For all other items, the time frame for disclosure is the past 36 months.

		**Name all entities with whom you have this relationship or indicate none (add rows as needed)**	**Specifications/Comments (e.g., if payments were made to you or to your institution)**
		**Time frame: Since the initial planning of the work**	
1	All support for the present manuscript (e.g., funding, provision of study materials, medical writing, article processing charges, etc.) **No time limit for this item.**	____ None<br>Rockefeller University Hospital	Pilot grant
		National Institute of General Medical Sciences	T32GM007739
		National Center for Advancing Translational Sciences	UL1TR001866
		National Cancer Institute	P30CA008748
		American Cancer Society	133831-CSDG-19-117-01-CPHPS
		American Diabetes Association	1-17-ACE-17
		private donor (Memorial Sloan Kettering Cancer Center	
		**Time frame: past 36 months**	
2		_x___ None	

	Grants or contracts from any entity (if not indicated in item #1 above).		
3	Royalties or licenses	_x___ None	
4	Consulting fees	_x___ None	
5	Payment or honoraria for lectures, presentations, speakers bureaus, manuscript writing or educational events	_x___ None	
6	Payment for expert testimony	_x___ None	
7	Support for attending meetings and/or travel	_x___ None	
8	Patents planned, issued or pending	_x___ None	
9	Participation on a Data Safety Monitoring Board or Advisory Board	_x___ None	
10	Leadership or fiduciary role in other board, society, committee or advocacy group, paid or unpaid	_x___ None	
11	Stock or stock options	_x___ None	
12	Receipt of equipment, materials, drugs, medical writing, gifts or other services	_x___ None	
13	Other financial or non-financial interests	_x___ None	

Please place an "X" next to the following statement to indicate your agreement:

_x_ I certify that I have answered every question and have not altered the wording of any of the questions on this form.

ICMJE DISCLOSURE FORM

Date:__9/1/21____
Your Name:_Thomas Carroll____
Manuscript Title: Therapeutic abdominal radiation during childhood induces chronic adipose tissue dysfunction
Manuscript number (if known): 153586-JCI-CMED-PRESUB

In the interest of transparency, we ask you to disclose all relationships/activities/interests listed below that are related to the content of your manuscript. "Related" means any relation with for-profit or not-for-profit third parties whose interests may be affected by the content of the manuscript. Disclosure represents a commitment to transparency and does not necessarily indicate a bias. If you are in doubt about whether to list a relationship/activity/interest, it is preferable that you do so.

The following questions apply to the author's relationships/activities/interests as they relate to the current manuscript only.

The author's relationships/activities/interests should be defined broadly. For example, if your manuscript pertains to the epidemiology of hypertension, you should declare all relationships with manufacturers of antihypertensive medication, even if that medication is not mentioned in the manuscript.

In item #1 below, report all support for the work reported in this manuscript without time limit. For all other items, the time frame for disclosure is the past 36 months.

		**Name all entities with whom you have this relationship or indicate none (add rows as needed)**	**Specifications/Comments (e.g., if payments were made to you or to your institution)**
		**Time frame: Since the initial planning of the work**	
1	All support for the present manuscript (e.g., funding, provision of study materials, medical writing, article processing charges, etc.) **No time limit for this item.**	____ None<br>Rockefeller University Hospital	Pilot grant
		National Institute of General Medical Sciences	T32GM007739
		National Center for Advancing Translational Sciences	UL1TR001866
		National Cancer Institute	P30CA008748
		American Cancer Society	133831-CSDG-19-117-01-CPHPS
		American Diabetes Association	1-17-ACE-17
		private donor (Memorial Sloan Kettering Cancer Center	
		**Time frame: past 36 months**	
2		_x___ None	

	Grants or contracts from any entity (if not indicated in item #1 above).		
3	Royalties or licenses	_x___ None	
4	Consulting fees	_x___ None	
5	Payment or honoraria for lectures, presentations, speakers bureaus, manuscript writing or educational events	_x___ None	
6	Payment for expert testimony	_x___ None	
7	Support for attending meetings and/or travel	_x___ None	
8	Patents planned, issued or pending	_x___ None	
9	Participation on a Data Safety Monitoring Board or Advisory Board	_x___ None	
10	Leadership or fiduciary role in other board, society, committee or advocacy group, paid or unpaid	_x___ None	
11	Stock or stock options	_x___ None	
12	Receipt of equipment, materials, drugs, medical writing, gifts or other services	_x___ None	
13	Other financial or non-financial interests	_x___ None	

Please place an "X" next to the following statement to indicate your agreement:

_x_ I certify that I have answered every question and have not altered the wording of any of the questions on this form.

ICMJE DISCLOSURE FORM

Date:__9/1/21____
Your Name:_Ji-Dung Luo____
Manuscript Title: Therapeutic abdominal radiation during childhood induces chronic adipose tissue dysfunction
Manuscript number (if known): 153586-JCI-CMED-PRESUB

In the interest of transparency, we ask you to disclose all relationships/activities/interests listed below that are related to the content of your manuscript. "Related" means any relation with for-profit or not-for-profit third parties whose interests may be affected by the content of the manuscript. Disclosure represents a commitment to transparency and does not necessarily indicate a bias. If you are in doubt about whether to list a relationship/activity/interest, it is preferable that you do so.

The following questions apply to the author's relationships/activities/interests as they relate to the current manuscript only.

The author's relationships/activities/interests should be defined broadly. For example, if your manuscript pertains to the epidemiology of hypertension, you should declare all relationships with manufacturers of antihypertensive medication, even if that medication is not mentioned in the manuscript.

In item #1 below, report all support for the work reported in this manuscript without time limit. For all other items, the time frame for disclosure is the past 36 months.

		**Name all entities with whom you have this relationship or indicate none (add rows as needed)**	**Specifications/Comments (e.g., if payments were made to you or to your institution)**
		**Time frame: Since the initial planning of the work**	
1	All support for the present manuscript (e.g., funding, provision of study materials, medical writing, article processing charges, etc.) **No time limit for this item.**	____ None<br>Rockefeller University Hospital	Pilot grant
		National Institute of General Medical Sciences	T32GM007739
		National Center for Advancing Translational Sciences	UL1TR001866
		National Cancer Institute	P30CA008748
		American Cancer Society	133831-CSDG-19-117-01-CPHPS
		American Diabetes Association	1-17-ACE-17
		private donor (Memorial Sloan Kettering Cancer Center	
		**Time frame: past 36 months**	
2		_x___ None	

	Grants or contracts from any entity (if not indicated in item #1 above).		
3	Royalties or licenses	_x___ None	
4	Consulting fees	_x___ None	
5	Payment or honoraria for lectures, presentations, speakers bureaus, manuscript writing or educational events	_x___ None	
6	Payment for expert testimony	_x___ None	
7	Support for attending meetings and/or travel	_x___ None	
8	Patents planned, issued or pending	_x___ None	
9	Participation on a Data Safety Monitoring Board or Advisory Board	_x___ None	
10	Leadership or fiduciary role in other board, society, committee or advocacy group, paid or unpaid	_x___ None	
11	Stock or stock options	_x___ None	
12	Receipt of equipment, materials, drugs, medical writing, gifts or other services	_x___ None	
13	Other financial or non-financial interests	_x___ None	

Please place an “X” next to the following statement to indicate your agreement:

_x_ I certify that I have answered every question and have not altered the wording of any of the questions on this form.

ICMJE DISCLOSURE FORM

Date:__9/1/21____
Your Name:_Emily Tonorezos____
Manuscript Title: Therapeutic abdominal radiation during childhood induces chronic adipose tissue dysfunction
Manuscript number (if known): 153586-JCI-CMED-PRESUB

In the interest of transparency, we ask you to disclose all relationships/activities/interests listed below that are related to the content of your manuscript. "Related" means any relation with for-profit or not-for-profit third parties whose interests may be affected by the content of the manuscript. Disclosure represents a commitment to transparency and does not necessarily indicate a bias. If you are in doubt about whether to list a relationship/activity/interest, it is preferable that you do so.

The following questions apply to the author's relationships/activities/interests as they relate to the current manuscript only.

The author's relationships/activities/interests should be defined broadly. For example, if your manuscript pertains to the epidemiology of hypertension, you should declare all relationships with manufacturers of antihypertensive medication, even if that medication is not mentioned in the manuscript.

In item #1 below, report all support for the work reported in this manuscript without time limit. For all other items, the time frame for disclosure is the past 36 months.

		**Name all entities with whom you have this relationship or indicate none (add rows as needed)**	**Specifications/Comments (e.g., if payments were made to you or to your institution)**
		**Time frame: Since the initial planning of the work**	
1	All support for the present manuscript (e.g., funding, provision of study materials, medical writing, article processing charges, etc.) **No time limit for this item.**	____ None<br>Rockefeller University Hospital	Pilot grant
		National Institute of General Medical Sciences	T32GM007739
		National Center for Advancing Translational Sciences	UL1TR001866
		National Cancer Institute	P30CA008748
		American Cancer Society	133831-CSDG-19-117-01-CPHPS
		American Diabetes Association	1-17-ACE-17
		private donor (Memorial Sloan Kettering Cancer Center	
		**Time frame: past 36 months**	
2		_x___ None	

	Grants or contracts from any entity (if not indicated in item #1 above).		
3	Royalties or licenses	_x___ None	
4	Consulting fees	_x___ None	
5	Payment or honoraria for lectures, presentations, speakers bureaus, manuscript writing or educational events	_x___ None	
6	Payment for expert testimony	_x___ None	
7	Support for attending meetings and/or travel	_x___ None	
8	Patents planned, issued or pending	_x___ None	
9	Participation on a Data Safety Monitoring Board or Advisory Board	_x___ None	
10	Leadership or fiduciary role in other board, society, committee or advocacy group, paid or unpaid	_x___ None	
11	Stock or stock options	_x___ None	
12	Receipt of equipment, materials, drugs, medical writing, gifts or other services	_x___ None	
13	Other financial or non-financial interests	_x___ None	

Please place an "X" next to the following statement to indicate your agreement:

_x_ I certify that I have answered every question and have not altered the wording of any of the questions on this form.

ICMJE DISCLOSURE FORM

Date:__9/1/21____
Your Name:_Danielle Novetsky Friedman____
Manuscript Title: Therapeutic abdominal radiation during childhood induces chronic adipose tissue dysfunction
Manuscript number (if known): 153586-JCI-CMED-PRESUB

In the interest of transparency, we ask you to disclose all relationships/activities/interests listed below that are related to the content of your manuscript. "Related" means any relation with for-profit or not-for-profit third parties whose interests may be affected by the content of the manuscript. Disclosure represents a commitment to transparency and does not necessarily indicate a bias. If you are in doubt about whether to list a relationship/activity/interest, it is preferable that you do so.

The following questions apply to the author's relationships/activities/interests as they relate to the <u>current manuscript only</u>.

The author's relationships/activities/interests should be <u>defined broadly</u>. For example, if your manuscript pertains to the epidemiology of hypertension, you should declare all relationships with manufacturers of antihypertensive medication, even if that medication is not mentioned in the manuscript.

In item #1 below, report all support for the work reported in this manuscript without time limit. For all other items, the time frame for disclosure is the past 36 months.

		Name all entities with whom you have this relationship or indicate none (add rows as needed)	Specifications/Comments (e.g., if payments were made to you or to your institution)
		**Time frame: Since the initial planning of the work**	
1	All support for the present manuscript (e.g., funding, provision of study materials, medical writing, article processing charges, etc.) **No time limit for this item.**	____ None<br>Rockefeller University Hospital	Pilot grant
		National Institute of General Medical Sciences	T32GM007739
		National Center for Advancing Translational Sciences	UL1TR001866
		National Cancer Institute	P30CA008748
		American Cancer Society	133831-CSDG-19-117-01-CPHPS
		American Diabetes Association	1-17-ACE-17
		Private donor (Memorial Sloan Kettering Cancer Center)	
		**Time frame: past 36 months**	
2		____ None	

	Grants or contracts from any entity (if not indicated in item #1 above).	National Cancer Institute	U01 CA246659-A01
		Sohn Foundation	grant
		Society of MSK Research	Grant
3	Royalties or licenses	_x___ None	
4	Consulting fees	_x___ None	
5	Payment or honoraria for lectures, presentations, speakers bureaus, manuscript writing or educational events	UpToDate	2014-Present
		ENDO2021: Annual Meeting of the Endocrine Society	March 2021
6	Payment for expert testimony	_x___ None	
7	Support for attending meetings and/or travel	Agence Nationale de la Recherche (ANR), French Cohortes, Jury member	09/16/2019-09/18/2019 (Travel to Paris, France)
8	Patents planned, issued or pending	_x___ None	
9	Participation on a Data Safety Monitoring Board or Advisory Board	_x___ None	
10	Leadership or fiduciary role in other board, society, committee or advocacy group, paid or unpaid	_x___ None	
11	Stock or stock options	_x___ None	
12	Receipt of equipment, materials, drugs, medical writing, gifts or other services	_x___ None	
13	Other financial or non-financial interests	_x___ None	

Please place an "X" next to the following statement to indicate your agreement:

_x_ I certify that I have answered every question and have not altered the wording of any of the questions on this form.

ICMJE DISCLOSURE FORM

Date:__9/1/21____
Your Name:_Paul Cohen____
Manuscript Title: Therapeutic abdominal radiation during childhood induces chronic adipose tissue dysfunction
Manuscript number (if known): 153586-JCI-CMED-PRESUB

In the interest of transparency, we ask you to disclose all relationships/activities/interests listed below that are related to the content of your manuscript. "Related" means any relation with for-profit or not-for-profit third parties whose interests may be affected by the content of the manuscript. Disclosure represents a commitment to transparency and does not necessarily indicate a bias. If you are in doubt about whether to list a relationship/activity/interest, it is preferable that you do so.

The following questions apply to the author's relationships/activities/interests as they relate to the current manuscript only.

The author's relationships/activities/interests should be defined broadly. For example, if your manuscript pertains to the epidemiology of hypertension, you should declare all relationships with manufacturers of antihypertensive medication, even if that medication is not mentioned in the manuscript.

In item #1 below, report all support for the work reported in this manuscript without time limit. For all other items, the time frame for disclosure is the past 36 months.

		**Name all entities with whom you have this relationship or indicate none (add rows as needed)**	**Specifications/Comments (e.g., if payments were made to you or to your institution)**
		**Time frame: Since the initial planning of the work**	
1	All support for the present manuscript (e.g., funding, provision of study materials, medical writing, article processing charges, etc.) **No time limit for this item.**	____ None<br>Rockefeller University Hospital	Pilot grant
		National Institute of General Medical Sciences	T32GM007739
		National Center for Advancing Translational Sciences	UL1TR001866
		National Cancer Institute	P30CA008748
		American Cancer Society	133831-CSDG-19-117-01-CPHPS
		American Diabetes Association	1-17-ACE-17
		private donor (Memorial Sloan Kettering Cancer Center	
		**Time frame: past 36 months**	
2		_x___ None	

	Grants or contracts from any entity (if not indicated in item #1 above).		
3	Royalties or licenses	_x___ None	
4	Consulting fees	_x___ None	
5	Payment or honoraria for lectures, presentations, speakers bureaus, manuscript writing or educational events	_x___ None	
6	Payment for expert testimony	_x___ None	
7	Support for attending meetings and/or travel	_x___ None	
8	Patents planned, issued or pending	_x___ None	
9	Participation on a Data Safety Monitoring Board or Advisory Board	_x___ None	
10	Leadership or fiduciary role in other board, society, committee or advocacy group, paid or unpaid	_x___ None	
11	Stock or stock options	_x___ None	
12	Receipt of equipment, materials, drugs, medical writing, gifts or other services	_x___ None	
13	Other financial or non-financial interests	_x___ None	

Please place an "X" next to the following statement to indicate your agreement:

_x_ I certify that I have answered every question and have not altered the wording of any of the questions on this form.

STROBE Statement—Checklist of items that should be included in reports of ***cohort studies***

	Item No	Recommendation
**Title and abstract**	1	(*a*) Indicate the study's design with a commonly used term in the title or the abstract
		(*b*) Provide in the abstract an informative and balanced summary of what was done and what was found
**Introduction**		
Background/rationale	2	Explain the scientific background and rationale for the investigation being reported
Objectives	3	State specific objectives, including any prespecified hypotheses
**Methods**		
Study design	4	Present key elements of study design early in the paper
Setting	5	Describe the setting, locations, and relevant dates, including periods of recruitment, exposure, follow-up, and data collection
Participants	6	(*a*) Give the eligibility criteria, and the sources and methods of selection of participants. Describe methods of follow-up
		(*b*) For matched studies, give matching criteria and number of exposed and unexposed n/a
Variables	7	Clearly define all outcomes, exposures, predictors, potential confounders, and effect modifiers. Give diagnostic criteria, if applicable
Data sources/ measurement	8*	For each variable of interest, give sources of data and details of methods of assessment (measurement). Describe comparability of assessment methods if there is more than one group
Bias	9	Describe any efforts to address potential sources of bias
Study size	10	Explain how the study size was arrived at
Quantitative variables	11	Explain how quantitative variables were handled in the analyses. If applicable, describe which groupings were chosen and why
Statistical methods	12	(*a*) Describe all statistical methods, including those used to control for confounding
		(*b*) Describe any methods used to examine subgroups and interactions n/a
		(*c*) Explain how missing data were addressed
		(*d*) If applicable, explain how loss to follow-up was addressed n/a
		(*e*) Describe any sensitivity analyses n/a
**Results**		
Participants	13*	(*a*) Report numbers of individuals at each stage of study—eg numbers potentially eligible, examined for eligibility, confirmed eligible, included in the study, completing follow-up, and analysed
		(*b*) Give reasons for non-participation at each stage
		(*c*) Consider use of a flow diagram
Descriptive data	14*	(*a*) Give characteristics of study participants (eg demographic, clinical, social) and information on exposures and potential confounders
		(*b*) Indicate number of participants with missing data for each variable of interest
		(*c*) Summarise follow-up time (eg, average and total amount)
Outcome data	15*	Report numbers of outcome events or summary measures over time
Main results	16	(*a*) Give unadjusted estimates and, if applicable, confounder-adjusted estimates and their precision (eg, 95% confidence interval). Make clear which confounders were adjusted for and why they were included
		(*b*) Report category boundaries when continuous variables were categorized n/a
		(*c*) If relevant, consider translating estimates of relative risk into absolute risk for a meaningful time period n/a

Other analyses	17	Report other analyses done—eg analyses of subgroups and interactions, and sensitivity analyses
**Discussion**		
Key results	18	Summarise key results with reference to study objectives
Limitations	19	Discuss limitations of the study, taking into account sources of potential bias or imprecision. Discuss both direction and magnitude of any potential bias
Interpretation	20	Give a cautious overall interpretation of results considering objectives, limitations, multiplicity of analyses, results from similar studies, and other relevant evidence
Generalisability	21	Discuss the generalisability (external validity) of the study results
**Other information**		
Funding	22	Give the source of funding and the role of the funders for the present study and, if applicable, for the original study on which the present article is based

*Give information separately for exposed and unexposed groups.

Note: An Explanation and Elaboration article discusses each checklist item and gives methodological background and published examples of transparent reporting. The STROBE checklist is best used in conjunction with this article (freely available on the Web sites of PLoS Medicine at http://www.plosmedicine.org/, Annals of Internal Medicine at http://www.annals.org/, and Epidemiology at http://www.epidem.com/). Information on the STROBE Initiative is available at http://www.strobe-statement.org.
